# Impact of Side Chains of Conjugated Polymers on Electronic Structure: A Case Study

**DOI:** 10.3390/polym11050870

**Published:** 2019-05-13

**Authors:** Clemens Matt, Florian Lombeck, Michael Sommer, Till Biskup

**Affiliations:** 1 Institut für Physikalische Chemie, Albert-Ludwigs-Universität Freiburg, Albertstraße 21, 79104 Freiburg, Germany; clemens.matt@physchem.uni-freiburg.de; 2 Institut für Makromolekulare Chemie, Albert-Ludwigs-Universität Freiburg, Stefan-Meier-Straße 31, 79104 Freiburg, Germany; Florian.Lombeck@Hahn-Schickard.de (F.L.); michael.sommer@chemie.tu-chemnitz.de (M.S.)

**Keywords:** side chains, electronic structure, triplet states, electron paramagnetic resonance, delocalization

## Abstract

Processing from solution is a crucial aspect of organic semiconductors, as it is at the heart of the promise of easy and inexpensive manufacturing of devices. Introducing alkyl side chains is an approach often used to increase solubility and enhance miscibility in blends. The influence of these side chains on the electronic structure, although highly important for a detailed understanding of the structure-function relationship of these materials, is still barely understood. Here, we use time-resolved electron paramagnetic resonance spectroscopy with its molecular resolution to investigate the role of alkyl side chains on the polymer PCDTBT and a series of its building blocks with increasing length. Comparing our results to the non-hexylated compounds allows us to distinguish four different factors determining exciton delocalization. Detailed quantum-chemical calculations (DFT) allows us to further interpret our spectroscopic data and to relate our findings to the molecular geometry. Alkylation generally leads to more localized excitons, most prominent only for the polymer. Furthermore, singlet excitons are more delocalized than the corresponding triplet excitons, despite the larger dihedral angles within the backbone found for the singlet-state geometries. Our results show TREPR spectroscopy of triplet excitons to be well suited for investigating crucial aspects of the structure-function relationship of conjugated polymers used as organic semiconductors on a molecular basis.

## 1. Introduction

Semiconductors have revolutionized our way of life and are currently ubiquitous materials for various devices and applications. However, most of these devices still consist of inorganic compounds, mostly silicon. While robust, these inorganic semiconducting devices are rather inflexible and limit the possible fields of application. Organic semiconductors, i.e., semiconductors based on organic molecules, mostly conjugated polymers, are very promising candidates for dramatically changing the way we apply the devices built upon them [1,2,3]. The big advantages of organic semiconductors over their more conventional inorganic counterparts are their mechanical flexibility [4,5,6,7], simple and inexpensive processing from solution [8], and variability due to well-developed protocols of synthetic chemistry. This renders wearable electronics [9,10], as well as large-area electronic devices [11] and flexible displays [12] viable, to name just a few potential applications.

While organic semiconductors still lag behind their inorganic counterparts in many respects, they have gained considerable interest in both academia and industry [13,14,15] and are now even competitive in some areas of electronics [16,17]. Potential and real applications range from Organic Photovoltaics (OPV) [18,19], Organic Light-Emitting Diodes (OLEDs) [20], and Organic Field-Effect Transistors (OFETs) [21] to sensors [2,22]. Key for their successful application is a fundamental understanding of the structure-function relationship of these materials. For OPV materials, two key aspects have been identified that can be extended to other applications as well: morphology [23] and electronic structure [24]. Many different methods are available to probe the morphology of conjugated polymers, at least in films. Recently, we demonstrated (time-resolved) Electron Paramagnetic Resonance (EPR) spectroscopy [25,26,27] to be able to probe both film [28] and solution [29] morphology with molecular resolution. In contrast to morphology, direct access to the electronic structure of conjugated polymers is much more difficult to achieve. Spectroscopic tools with molecular resolution capable of directly probing the electronic structure of the polymer are of high demand.

**Figure 1:**
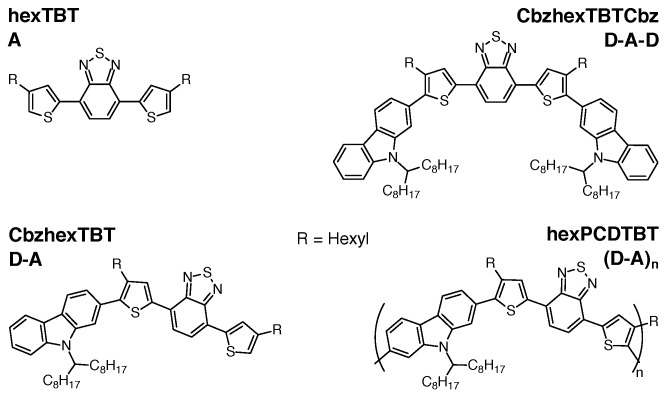
Chemical structure of hexPCDTBT and its building blocks. The polymer repeat unit Carbazole (Cbz)-hexTBT forms a push-pull system comprising a Carbazole (Cbz) as donor and a hexylated Dithienyl-Benzothiadiazole (TBT) unit as the acceptor, the latter being itself a push-pull system. For simplicity, the TBT acceptor unit will be abbreviated with “A” and the Cbz donor unit with “D” hereafter.

Key to the simple and inexpensive manufacturing of organic semiconductor devices is solution processing of the components [30]. Hence, their solubility is of particular importance. To control both molecular weight and dispersity, premature precipitation of the polymer synthesized should be avoided. Introducing linear or branched alkyl side chains is an approach often followed to enhance solubility [31,32,33,34,35,36]. However, its potential impact on the electronic structure of the backbone is neither trivial nor well-understood. Generally, investigating the building blocks of increasing backbone length has been proven useful to gain deeper insight into the electronic structure of conjugated polymers [37,38,39]. In this study, we apply this approach to the hexylated version of the common copolymer PCDTBT (poly[*N*-9${}^{\prime }$-heptadecanyl-2,7-carbazole-*alt*-5,5-(4${}^{\prime }$,7${}^{\prime }$-di-2-thienyl-2${}^{\prime }$,1${}^{\prime }$,3${}^{\prime }$-benzothiadiazole)]) [40,41], investigating both the polymer hexPCDTBT, as well as three building blocks with increasing backbone length (Figure 1). The polymer repeat unit consists of a Carbazole (Cbz) moiety acting as Donor (D) and a hexylated dithienyl-benzothiadiazole (hexTBT) moiety serving as Acceptor (A). We note that hexTBT in itself is a push-pull system comprised of the central benzothiadiazole acceptor and the two flanking (hexylated) thiophene donors. For simplicity, we will refer to Cbz and hexTBT as Donor (D) and Acceptor (A) hereafter.

PCDTBT is well known for its high power conversion efficiencies up to 7–8% [42] combined with robustness and air-stability over a long time [43,44,45,46,47], but also for its tendency to form carbazole homocouplings [48]. Recently, we demonstrated this polymer to form rather ordered structures in drop-cast films [28]. This came quite to our surprise, as the polymer is known for its amorphous morphology [49], not readily forming crystals [50]. Furthermore, an overall face-on orientation with respect to the substrate was deduced, which is advantageous for charge carrier injection or extraction in OLED or OPV devices, respectively [51,52,53,54]. Additionally, we investigated the impact of side chains on the film morphology using a series of PCDTBT polymers with increasing degree of alkyl side chains, allowing us to distinguish between effects on electronic structure and morphology [55].

Introducing additional hexyl side chains at the TBT unit has been shown to increase backbone torsion and luminescence, effectively converting it from a material for photovoltaics to one for OLEDs [56]. Regarding the thermal properties, additional hexyl side chains reduce the glass transition temperature $T_{g}$ from 127 ${}^{\circ }$C (PCDTBT) to 93 ${}^{\circ }$C for the fully-hexylated version hexPCDTBT. At the same time, a sometimes very weak melting point of PCDTBT at 235 ${}^{\circ }$C vanishes upon hexylation [56]. Here, we combine synthetic chemistry giving access to a series of building blocks of different lengths with Time-Resolved EPR (TREPR) spectroscopy allowing for molecular resolution and detailed mapping of the electronic structure and extended quantum-chemical (DFT) calculations. Comparing our results on the hexylated molecules with those obtained earlier for the non-hexylated ones [39] allows us to map the influence of the additional alkyl chains on the electronic structure of the molecules with unprecedented accuracy. Given the wide-spread use of branched or linear alkyl side chains to enhance solubility and miscibility [33,34,35,36], the results obtained are highly relevant far beyond the actual polymer system investigated here.

## 2. Materials and Methods

Synthesis

All molecules and materials were synthesized according to published procedures as described in detail elsewhere. hexTBT was synthesized according to [57]. CbzhexTBT was synthesized according to [58]. CbzhexTBTCbz was synthesized according to [59]. hexPCDTBT was synthesized according to [56].

Optical spectroscopy

All samples were dissolved in *o*-dichlorobenzene. Absorption spectra were recorded using a commercial UV-Vis spectrometer (Shimadzu UV-2450, UV-1601PC) in combination with the corresponding software (UV Probe Version 3.42, all Shimadzu, Kyoto). Measurements at ambient temperatures were performed with standard path length cuvettes (1 cm). Temperature series were recorded using a cryostat (Optistat DN2) in combination with a temperature controller (MercuryITC, both Oxford Instruments, Abingdon) and using liquid nitrogen as a coolant. These measurements were performed using 1-mm path length cuvettes.

EPR Instrumentation

All samples were dissolved in *o*-dichlorobenzene. All TREPR experiments were performed at 80 K using a setup described previously [28,58]. Optical excitation at the respective wavelengths was carried out using an Optical Parametric Oscillator (OPO) pumped by a Nd:YAG laser. The repetition rate of the laser was set to 10 Hz and the final pulse energy (after the OPO) to 1 mJ. Further experimental parameters (except where explicity given) were as follows: Microwave frequency, 9.700 GHz, microwave power: 2 mW (20 dB attenuation, source power 200 mW), frequency-mixer detection, video amplifier set to a 42-dB amplification and a 25-MHz bandwidth, between 850 and 1400 averages per point.

Spectral simulations

All simulations of triplet spectra were performed using the pepper routine from the EasySpin software package [60] available for MATLAB^®^ (MathWorks, Natick, MA). Details of both the simulations and the fitting procedure are given in the Supplementary Materials.

DFT calculations

All calculations were performed using ORCA 3.0.3 [61] using the BP86 [62,63] and B3LYP [64,65] functionals and the Def2-SVP [66] and 6-31G** [67,68] basis sets, respectively. For ***D*** tensor calculations, the EPR-II basis set [69] was used. The solvent has been accounted for by the COSMO model [70]. Initial geometries of the molecules were created using Avogadro 1.1.1 [71]. Spin density plots were created using UCSF Chimera 1.11.2 [72]. Extracting dihedral angles and tensor geometries from the calculations was performed using MATLAB routines written specifically for this purpose.

## 3. Results

### 3.1. Absorption Spectra

**Figure 2:**
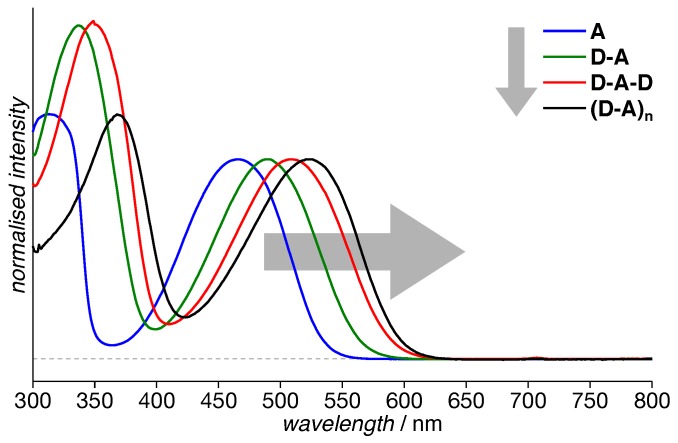
Absorption spectra of hexPCDTBT and its building blocks. Each spectrum consists of two bands, a π–π* band (mostly) in the near-UV region and a prominent band in the visible region, assigned to the partial Charge-Transfer character of the molecule and hence termed CT band. Obviously, the CT band increasingly shifts towards greater wavelengths with increasing length of the backbone. For easier comparison, spectra have been normalized to the same CT band height.

For all building blocks and the polymer, steady-state absorption spectra have been recorded at room temperature in solution (Figure 2). For each of the molecules, the spectrum consisted of a prominent absorption band in the visible region, due to its (partial) Charge-Transfer (CT) character usually termed the CT band, and a second band in the near-UV region that can be ascribed predominantly to a π–π* transition [37]. Obviously, the CT band increasingly shifts towards larger wavelengths with increasing length of the backbone. Whereas this red-shift is nearly identical for going from **A** to **D-A** and from **D-A** to **D-A-D**, it is dramatically reduced for proceeding from **D-A-D** to the polymer, **(D-A)_n_**.

Additionally, we note that the intensity of the π–π* transition is always higher than the CT band. Furthermore, there is no trend proceeding from **A** to **(D-A)_n_**. Rather, for **A** and **(D-A)_n_**, the π–π* transition is only slightly stronger than the CT band, about 20%, whereas for **D-A** and **D-A-D**, the π–π* transition is more than 1.5× more intense. It is tempting to assign these differences to a different degree of charge-transfer character of the (singlet) exciton in the respective molecule.

Besides giving insight into the electronic structure and delocalization of the singlet exciton, these steady-state absorption spectra are useful to determine the excitation wavelength for TREPR spectroscopy. Due to the fast spin relaxation of the strongly-coupled electron spins within a triplet state, TREPR spectra of these molecules need to be recorded in solid state under cryogenic conditions (see details below and in the Supplementary Materials). To rule out aggregation taking place upon cooling and to impact our interpretation of the EPR data, we recorded temperature-dependent absorption spectra for each of the four compounds investigated (see the Supplementary Materials for actual spectra). Due to the solvent, *o*-dichlorobenzene (*o*-DCB), not forming a transparent glass upon freezing, these spectra could only be recorded down to the freezing point. The choice of solvent can quite dramatically influence the behavior of the molecules [29]. From the temperature-dependent spectra, we can clearly rule out any aggregation taking place upon slow cooling. Hence, we are pretty sure the sample morphology in solution was preserved, i.e., fully-solvated molecules, upon shock-freezing our EPR samples prior to measuring.

### 3.2. TREPR Spectra

**Figure 3:**
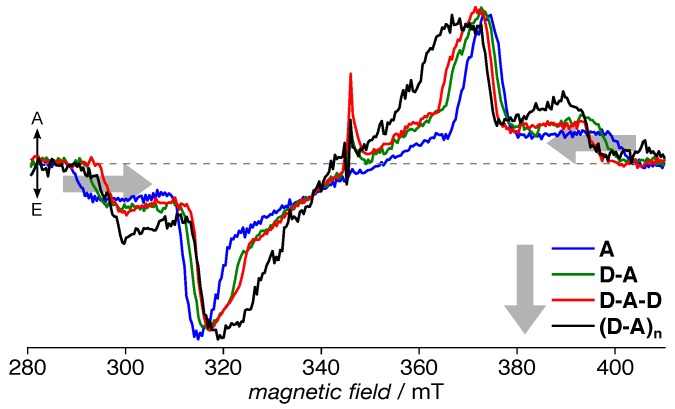
TREPR spectra of hexPCDTBT and its building blocks. Each sample has been excited in the respective CT band absorption maximum, cf. Figure 2. Obviously, the overall spectral width decreases with increasing length of the backbone. Spectra are averages over 200 ns, centered about 500 ns after the laser flash (in the maximum of the signal), and have been normalized to same absolute area under the respective curves. Each spectrum could be reproduced by spectral simulations taking a single triplet species into account (cf. Figure 4). For full two-dimensional datasets, see the Supplementary Materials.

Upon excitation at the maximum of the respective CT band, all compounds investigated here readily showed EPR spectra that can be clearly and unequivocally assigned to a single triplet species each (cf. Figure 3). One particular strength of EPR spectroscopy in general and TREPR spectroscopy in particular is not only its exclusive sensitivity to paramagnetic states, but also the clear distinction possible between triplet states and coulombically-bound polaron pairs, often termed charge-transfer complexes or radical pairs [25,73]. Both states consist of two unpaired electron spins interacting with each other via dipolar and exchange coupling. The interaction strength strongly depends on the average distance *r* between the two electron spins. In the case of a triplet state with the two electrons residing on the same chromophore, hence in close vicinity, the resulting EPR spectrum is usually entirely dominated by the dipolar interaction that can directly be estimated from the spectra (see the Supplementary Materials for details). A radical pair with its much larger separation of the two electron spins exhibited a much weaker dipolar and exchange interaction, the latter often negligible. Hence, its spectral width is dramatically reduced as compared to a triplet state [25,73].

The spin Hamilton operator for an organic triplet state in the presence of an external magnetic field can be written as:

(1) $$\begin{array}{cc} \hat{H} & =g_{e}\beta_{e}B^{T}\;\;\cdot \;\hat{S}+{\hat{S}}^{T}\;\;\cdot \;D\;\cdot \;\hat{S}. \end{array}$$

Here, the first term describes the electron Zeeman interaction and the second term the Zero-Field Splitting (ZFS) interaction with the corresponding interaction tensor:

(2) $$\begin{array}{cc} D & =\left(\begin{array}{ccc} -\frac{1}{3}D+E & 0 & 0\\ 0 & -\frac{1}{3}D-E & 0\\ 0 & 0 & \frac{2}{3}D \end{array}\right). \end{array}$$

In the frame of the ***D*** tensor, the second term can be rewritten in terms of the two scalar ZFS parameters *D* and *E*. The spin–spin contributions to these two parameters are defined as:

(3) $$\begin{array}{cc} D & =\frac{\;3D_{z}\;}{2}=\frac{3}{4}\left(\frac{\mu_{0}}{4\pi }\right){\left(g_{e}\beta_{e}\right)}^{2}\langle \frac{r^{2}-3z^{2}}{r^{5}}\rangle \;, \end{array}$$

(4) $$\begin{array}{cc} E & =\frac{D_{x}-D_{y}}{2}=\frac{3}{4}\left(\frac{\mu_{0}}{4\pi }\right){\left(g_{e}\beta_{e}\right)}^{2}\langle \frac{x^{2}-y^{2}}{r^{5}}\rangle . \end{array}$$

The angular brackets denote integration over the triplet state wave function, i.e., the spatial distribution of the two unpaired electrons of the triplet state. Different conventions can be found in the literature, and we followed here the convention used by EasySpin [60] for the order of the ***D*** tensor values, namely $|D_{z}\left|>\right|D_{y}\left|>\right|D_{x}|$, assuming, inter alia, |E|≤|D|/3 [74] and E/D>0. This is in contrast to [75] with respect to the order of $D_{x}$ and $D_{y}$. However, this affects only the assignment of the *x* and *y* axes of the ***D*** tensor, not the order of the triplet energy levels or the assignment of the populations to these levels depending on the sign of *D*. “The sign of *E* depends on the specific assignment of the axes *X* and *Y* and thus has no physical meaning except in terms of the convention that we have chosen” ([75], p. 167).

The small features visible in the center of some of the TREPR spectra are clearly not due to the triplet state. Their origin remains largely unknown, and different explanations have been brought forward in the past, ranging from photoinduced stable radicals, i.e., defects, to short-lived charge-transfer states, i.e., radical pairs, to species with higher spin multiplicity such as interacting triplet states. As they do not impair our tripet state simulations nor the interpretation of our data, they will not be discussed any further here.

**Figure 4:**
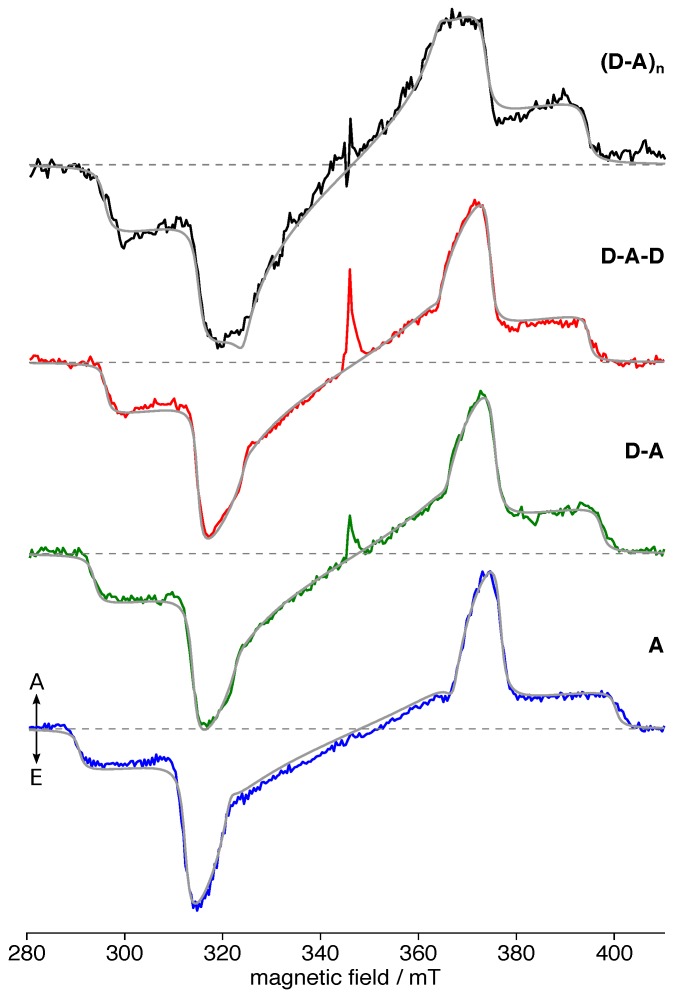
TREPR spectra of hexPCDTBT and its building blocks together with spectral simulations. Each spectrum could be reproduced by spectral simulations taking a single triplet species into account and shown as grey lines. For simulation parameters, cf. Table 1, for details of the fitting procedure see the Supplementary Materials. The small features in the center of the spectra at about 345 mT are not due to the triplet state and will not be accounted for here.

The TREPR spectra of each of the four compounds can be simulated taking into account a single triplet species each, as evident from the near-perfect fits (Figure 4). For simulation parameters, cf. Table 1. We note that each spectrum could be nicely reproduced taking into account only homogeneous (Lorentzian) line broadening, hinting at an overall very homogeneous environment of the exciton. Additionally, no population in the lowest-lying triplet sublevel, $p_{1}$, was necessary to reproduce the spectra. The overall trend of decreasing dipolar interaction with increasing backbone extent, as evident from the narrowing of the spectra (Figure 3), is reflected in the simulation parameters, as well. Furthermore, the triplet sublevel populations showed a trend of increasing $p_{2}$ with respect to $p_{3}$. However, no other consistent trends similar to the situation in the non-hexylated system [39] can be found. The rhombicity |E|/|D| increased slightly from **A** to **D-A-D**, but reduced again for **(D-A)_n_**. Whereas the overall spectral width and the |D| values were nearly identical for **D-A-D** and **(D-A)_n_**, the latter exhibited an overall somewhat different electronic structure, reflected in changes in the spectral shape. Nevertheless, the general spectral shape of all compounds remained rather similar, with an overall quite small rhombicity close to fully axial spectra, a small and only homogeneous line broadening, and a consistent order of the triplet sublevel populations with $p_{1}<p_{2}<p_{3}$.

Generally, the value of |D| can be assigned to the average distance between the two unpaired electron spins within the triplet state and therefore the triplet exciton delocalization, and |E| to its rhombicity, hence deviation from a fully-axial symmetry. Given the slightly different rhombicity for **D-A-D** and **(D-A)_n_**, the identical values for |D| within experimental error for these two compounds did not necessarily imply a fully-identical exciton delocalization. However, the trend already observed for the singlet exciton from the absorption spectra (Figure 2) was retained: Whereas a clear progression towards delocalization can be observed from **A** to **D-A-D**, the effect was much smaller for the polymer **(D-A)_n_**.

### 3.3. DFT Calculations

To gain further insight, a series of quantum-chemical calculations has been performed for each of the building blocks and two oligomer fragments with n=4 and n=7. Molecular geometries have been optimized both for singlet and triplet states, and for triplet states, the spin-density distribution, as well as the ***D*** tensors have been calculated. Furthermore, we compared two different functionals and basis sets.

First, for each of the building blocks and the oligomer fragments with n=4 and n=7, geometry optimizations have been performed for both singlet and triplet state and with two combinations of the functional and basis set, namely BP86/Def2-SVP and B3LYP/6-31G**. Note that the hexyl side chains have been fully accounted for in the calculations, whereas the branched alkyl chain attached to the nitrogen of the Cbz moiety has been omitted to save computational time. To validate this approach, we calculated geometries for the **D-A** fragment with and without branched alkyl chains on the Cbz moiety and found the dihedral angles between the aromatic planes of D and A to be mutually identical, in line with previous theoretical investigations obtained for the non-hexylated polymer [76]. For the spin-density distribution, four sets of calculations have been performed, with both combinations of the functional and basis set for both geometries. The combination fitting best to the experimental results is the one using the geometries optimized on the BP86/Def2-SVP level of theory and performing single-point calculations for the spin-density distribution with B3LYP/6-31G**. ***D*** tensors have been calculated as well for both geometries, but via single-point calculations performed on the B3LYP/EPR-II level of theory often used for calculating EPR parameters. In our previous study of non-hexylated PCDTBT and its building blocks [39], we have already shown the geometries obtained on the BP86/Def2-SVP level of theory to be more consistent with the experimental results. The same is true for the hexylated compounds studied here. Therefore, in the following, only the results from calculations based on these geometries are shown. For the results obtained for the other geometries, see the Supplementary Materials.

**Figure 5:**
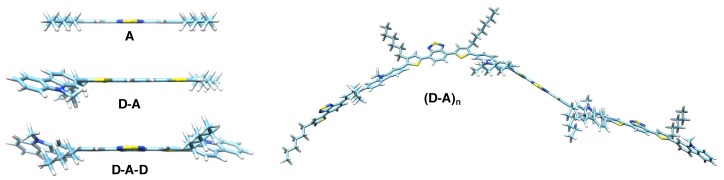
Side-view of the optimized geometries of the triplet states of hexPCDTBT and its building blocks. Geometries have been optimized for the triplet state on the theory level BP86/Def2-SVP. For a view perpendicular to the aromatic plane, cf. Figure 6. The D and A moieties in themselves are rather flat, whereas the aromatic planes of D and A moieties are tilted by substantial dihedral angles, respectively. For values of these dihedral angles, cf. Table 2.

Figure 5 gives a first impression of the geometries obtained for the triplet state. In any case, the acceptor moiety (hexTBT) in itself is planar, as well as the donor moiety (Cbz), and only between D and A, there is a quite substantial torsion, in line with previous analysis [56]. In order to obtain useful information of the planarity of the geometries, we calculated the dihedral angles between the aromatic planes of D and A, respectively (Table 2). For details of how these angles have been calculated, see the Supplementary Materials. Interestingly, for both combinations of functionals and basis sets (BP86/Def2-SVP and B3LYP/6-31G**), the geometries obtained for the triplet state were more planar as compared to those of the singlet state. This is in line with previous results obtained for an entirely different conjugated polymer, PNDIT2 [38]. Furthermore, we note that for the triplet state, the angles adjacent to the acceptor moiety carrying most of the spin density were smaller than average. This means that the triplet state locally planarizes the polymer backbone. To exclude possible boundary effects due to the hexTBT moiety carrying the maximum spin density in the fragment with n=4 being located towards one end of the chain, calculations for a fragment with n=7 have been performed as well. Note that the longer fragment with n=7 had a D moiety on both ends. Both polymer fragments showed a consistent behavior.

**Figure 6:**
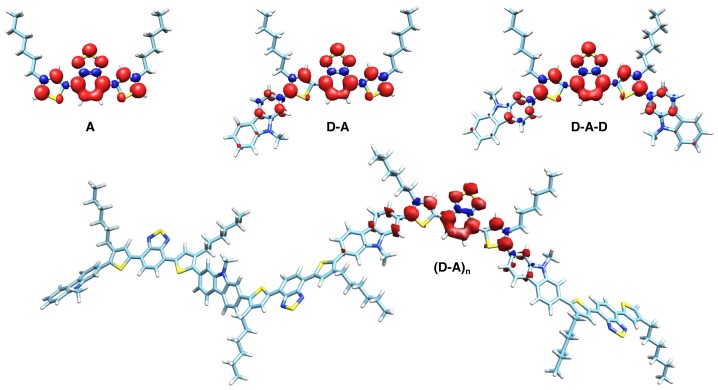
Spin-density distribution for the triplet states of hexPCDTBT and its building blocks. Geometries have been optimized for the triplet state on the theory level BP86/Def2-SVP and spin densities calculated on the theory level B3LYP/6-31G**. The latter have been displayed for a threshold level of ±0.002 (building blocks) and ±0.001 (polymer). Red denotes positive and blue negative spin density.

The spin density of the triplet states was centered on a single acceptor moiety for each of the building blocks and the polymer fragments (Figure 6), consistent with our previous results obtained for the non-hexylated polymer [39]. Hence, TBT seems to dominate the electronic structure of the polymer entirely, in line with the minor changes in the overall spectral shape of the TREPR data of the respective triplet states (Figure 4).

**Figure 7:**
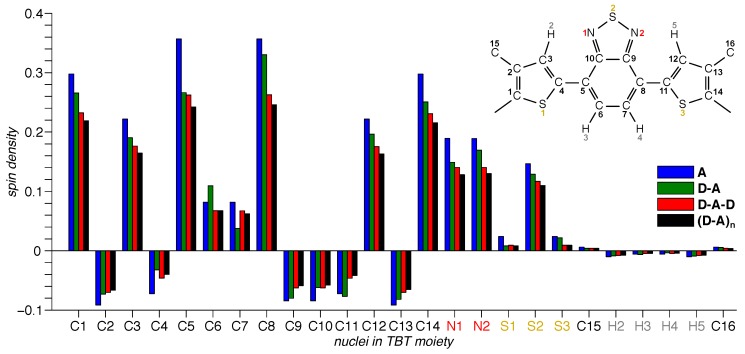
Quantitative analysis of the spin-density distribution for the triplet states of hexPCDTBT and its building blocks. Depicted are the values for the spin densities for each of the hexTBT atoms in the four fragments of different sizes displayed in Figure 6. The inset shows the numbering of the atoms used as axis labels. The hexyl chains are attached at positions C15 and C16. For the asymmetric building block, **D-A**, the D unit was attached to the left of the A unit, hence on the carbon atom labeled “C1”.

Whereas graphical representations of the spin densities as in Figure 6 provide an overall picture of their distribution upon a molecule, further analysis requires careful quantification of the information obtained. Therefore, we calculated the relative amount of spin density of the dominating acceptor unit, $\rho_{\mathrm{TBT}}$, for each of the fragments with varying backbone length. For actual values, cf. Table 3, and for details of how this information has been obtained, see the Supplementary Materials. Furthermore, we quantified the spin density on each of the atoms of the dominating acceptor unit (Figure 7). The latter allowed us to investigate in more detail the asymmetry introduced by adding a single donor moiety to one end of the acceptor. Note that in this case, the donor moiety was attached to the left of the acceptor moiety, hence on the carbon atom labeled “C1” in Figure 7.

**Figure 8:**
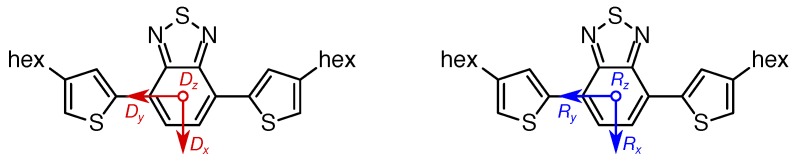
Orientation of the calculated ***D*** tensor within the TBT acceptor moiety and molecular reference frame. For each of the fragments investigated, the ***D*** tensor is basically oriented in the same way, as shown on the left. Assuming a right-handed coordinate system, the *z* component is pointing towards the paper plane. Only for the asymmetric repeat unit **D-A**, a slight deviation from the molecular reference frame ($R_{i}$ with i={x,y,z}, right) of a few degrees has been obtained from the DFT calculations. The deviation from the reference frame is given as three dihedral angles, α, β, and γ, for each of the three axes, *x*, *y*, and *z*, respectively. For actual values of these angles, see Table 3.

Although ***D*** tensors are known to be notoriously difficult to calculate using DFT methods, at least the calculated absolute values for the *D* and *E* parameter to deviate dramatically from experimental results, quantum-chemical calculations may well provide additional insight into the orientation of the ***D*** tensor within the molecular frame [77]. Hence, we calculated the ***D*** tensor for each of the compounds for both geometries (using the BP86/Def2-SVP and B3LYP/6-31G** level of theory, respectively) using B3LYP as the functional and EPR-II as the basis set. Interestingly, all calculated tensors exhibited a mutually-identical orientation within the molecular frame, with their *x* and *y* axes within the aromatic plane of the acceptor moiety and the *z* axis perpendicular to it. The *x* axis points perpendicular to the axis connecting thiophene-benzothiadiazole and the *y* axis along this connection (Figure 8). Assigning the ***D*** tensor axes is based on the usual convention $|D_{z}\left|>\right|D_{y}\left|>\right|D_{x}|$, assuming, inter alia, |E|≤|D|/3 [74] and E/D>0. Note that this convention follows EasySpin [60], but is in contrast to [75] with respect to the order of $D_{x}$ and $D_{y}$. However, this affects only the assignment of the *x* and *y* axes of the ***D*** tensor, not the order of the triplet energy levels or the assignment of the populations to the these levels depending on the sign of *D*. The molecular reference frame is given in Figure 8 (right), with the *x* and *y* axes as well within the aromatic plane of the acceptor moiety and the *z* axis perpendicular to it, accordingly. The deviations (dihedral angles) of the ***D*** tensor axes from the molecular reference frame are given in Table 3.

Whereas generally, the ***D*** tensor axes were collinear with the molecular reference frame as depicted in Figure 8, only for the asymmetric **D-A** building block, a slight tilt of the *x* and *y* axes by a few degrees towards the donor moiety can be seen. Whereas the angles given in Table 3 were calculated for the geometry obtained using the BP86/Def2-SVP level of theory, they were mutually identical for the geometry obtained using B3LYP/6-31G**. The same is true for the non-hexylated compounds for which the ***D*** tensors have been calculated, as well. Here, the deviation of the *x* and *y* axes from the molecular reference frame for the asymmetric repeat unit was slightly larger, amounting to about four degrees, each. See the Supplementary Materials for further details.

As can be seen from Table 3, as well, the calculated |D| values were about half of the size of those experimentally obtained. The opposite is true for the |E| values, where the calculations overestimated the parameter by about a factor of two. Hence, the rhombicity |E|/|D| of the calculated tensors dramatically deviated from those experimentally obtained. Given that the |D| and |E| values can be determined very accurately from the experimental EPR data, this clearly demonstrates the limits of the current approaches of DFT calculations for these types of parameters. At least the overall trend of decreasing |D| values for increasing fragment length is reflected in the calculated values. A smaller |D| value means a weakened dipolar interaction interpreted as increasing separation of the two unpaired electron spins of the triplet state and hence a larger delocalization of the exciton. The sign of the *D* values calculated using ORCA is always positive. Based on other studies [77] comparing in more detail experimentally-obtained signs of *D* with results from calculations, we are quite confident that assigning a positive sign to *D* is justified in our case, resulting in an oblate spin-density distribution. This allows assigning the populations $p_{1,2,3}$ to triplet energy levels $T_{x,y,z}$ using the conventions given above.

## 4. Discussion

Besides discussing the results described above, they will be compared to the results obtained in a previous study on the non-hexylated polymer and its building blocks [39]. This approach allows us to reveal the details of the impact the alkyl chains have on the electronic structure of the molecules and to distinguish the different aspects that are influenced, such as electronics and sterics. For a direct comparison of both spectra and parameters of the hexylated and non-hexylated system, the reader is referred to the Supplementary Materials.

### 4.1. Acceptor Dominates Electronic Structure

As obvious particularly from the TREPR spectra (Figure 4), the TBT acceptor moiety entirely dominates the electronic structure even of the polymer. Overall, the TREPR spectrum obtained for the polymer clearly resembles that of the acceptor alone, with only very minor changes in the overall shape and a narrower appearance due to increased delocalization of the exciton. This is in line with both the absorption spectra showing a dominating CT band in the visible range for all compounds investigated and our previous study on the non-hexylated polymer [39]. Hence, the same applies here, namely that referring to the polymer as a carbazole derivative, although chemically entirely correct, does not really reflect the situation in terms of its electronic structure.

DFT calculations of the spin-density distribution (Figure 6) further support the dominating role the acceptor moiety plays for the electronic structure of the polymer, as well as all the building blocks. Even for the polymer, about 85 percent of the total spin density resides on a single TBT moiety ($\rho_{\mathrm{TBT}}$ in Table 3). This is more than in case of the non-hexylated polymer and can be explained by the smaller delocalization of the triplet exciton on the hexylated polymer, as evident from comparing the |D| values and spectral widths. The detailed quantitative investigation of the spin density for each of the atoms of the acceptor moiety (Figure 7) reveals the Benzothiadiazole (BT) moiety to dominate within the TBT unit, carrying about half of the total spin density. Furthermore, this histogram reveals that for all compounds investigated, the spin density is distributed highly symmetrical upon the TBT unit, with one notable exception. The intrinsically-asymmetric polymer repeat unit, **D-A**, shows an asymmetric spin density pattern particularly for the central BT, with alternating increased and diminished spin density for adjacent atoms. As can be seen from Figure 6, the spin density advances to the flanking carbazole moieties, in line with an increased delocalization with extended backbone length.

### 4.2. Exciton Delocalization Extends with Backbone Length

**Figure 9:**
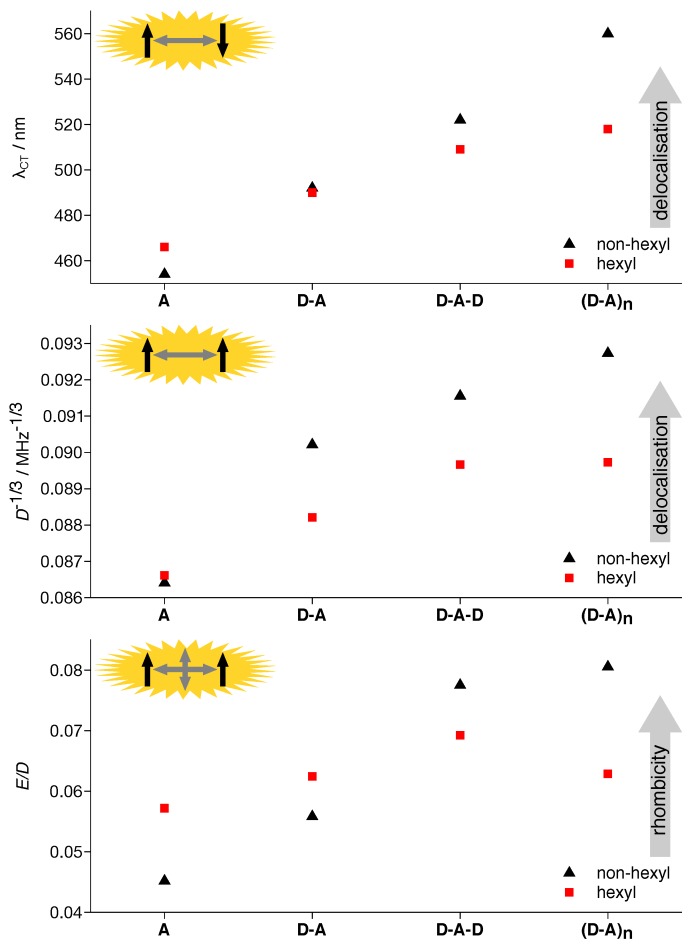
Comparison of the characteristics of singlet and triplet excitons for hexPCDTBT and PCDTBT and their respective building blocks. Delocalization of the singlet excitons (top) and triplet excitons (middle) follow overall the same trend, i.e., increasing delocalization with increasing backbone extent. In both cases, the relative differences were much larger for the non-hexylated compounds. For the rhombicity of the triplet exciton (bottom), a clear trend is only visible for the non-hexylated compounds, with rhombicity increasing with backbone extent.

Both singlet and triplet excitons exhibit an increasing delocalization with extended backbone length, as obvious from optical (Figure 2) and TREPR data (Figure 3) and the simulation parameters for the latter (Table 1). To help with extracting trends and with comparing the data obtained for the hexylated system with those of the non-hexylated system [39], the crucial parameters have been plotted in Figure 9. Whereas the absorption maximum of the CT band is a measure for the delocalization of the singlet exciton, the *D* parameter obtained by fitting simulations to the EPR spectra can be related to the spread of the triplet exciton. As *D* follows an inverse cubed distance dependence, $D^{-1/3}$ has been plotted.

A number of conclusions can be drawn immediately from the data presented in Figure 9. Whereas singlet and triplet exciton delocalization followed the same overall trend for both, non-hexylated and hexylated compounds, the delocalization of the triplet excitons was much more affected by the hexylation than that of the singlet excitons, with the delocalization of the bare acceptor unit being equal. As both, D and A moieties were in themselves pretty flat, the only difference between the hexylated and the non-hexylated compounds was the dihedral angle between the aromatic planes of D and A. Hence, we ascribed the overall stronger localization of the excitons for the hexylated polymer to the backbone torsion. Furthermore, this torsion seems to affect the delocalization of the triplet exciton much stronger than that of the singlet exciton.

The exciton delocalization on the bare acceptor unit deserves a special comment. Whereas for the singlet exciton, hexylation leads to a clearly visible red-shift of the CT band of 12 nm and thus an increased delocalization, the *D* values for the corresponding triplet exciton were nearly identical. We attributed the increased delocalization of the singlet exciton upon hexylation to the +I effect of the hexyl side chains. Furthermore, it seems to have a much stronger influence on the singlet exciton as compared to the triplet exciton. This is in line with the nearly negligible spin density residing on the carbon atoms (C16 and C16, Figure 7) of the hexyl side chains.

Additionally, while the non-hexylated compounds followed a monotonic trend with nearly identical slope for the exciton delocalization of both, singlet and triplet excitons, for the hexylated compounds, the increase in delocalization when proceeding from **D-A-D** to **(D-A)_n_** was clearly reduced. Particularly for the triplet exciton, the *D* values for **D-A-D** and **(D-A)_n_** were identical within experimental error. Nevertheless, the **D-A-D** fragment did not reflect the situation in the polymer. Whereas sharing a similar extent of the triplet exciton, both rhombicity and, more importantly, triplet sublevel populations were different, resulting in a clearly altered spectral shape (cf. Figure 4).

For the polymer, not only the band gap, but the HOMO level, as well, has been obtained experimentally in a previous study using Ultraviolet Photoelectron Spectroscopy (UPS) [56]. From these data, it is obvious that hexylation affects almost exclusively the HOMO level, but not the LUMO level.

### 4.3. Twisting the Backbone Reduces Curvature

A characteristic of the PCDTBT polymer is its flat and s-shaped backbone [78] that has been used to interpret the monotonic increase in rhombicity of the triplet exciton with increasing backbone length [39]. Interestingly, no such trend can be seen for the rhombicity of the spin-density distribution of the triplet excitons for the hexylated compounds. Directly comparing the rhombicity of hexylated and non-hexylated compounds (Figure 9, lower panel) shows that its values were higher for the hexylated compounds only for **A** and **D-A**, but smaller for **D-A-D** and **(D-A)_n_**. Additionally, the rhombicity slightly decreased again when proceeding from **D-A-D** to **(D-A)_n_**. We interpreted this in light of the sidechain-induced backbone torsion obvious from the geometry-optimized fragments (Table 2). Note that hexylation only introduces backbone torsion between adjacent D and A units, not within the A unit itself, which remains flat. Hence the sidechain-induced backbone torsion masks the backbone curvature dominating in the non-hexylated polymer. This is reflected in the rhombicity of PCDTBT being much larger than that of hexPCDTBT.

### 4.4. Triplet Excitons Planarize the Polymer Backbone

A very interesting trend can be deduced from the dihedral angles obtained from the optimized geometries (Table 2): The triplet excitons seem to locally planarize the polymer backbone. An overall more planar geometry for triplet-state geometries as compared to singlet-state geometries has been described before for a different polymer system, PNDIT2 [38]. The same seems true for the building blocks of hexylated PCDTBT investigated here. On average, the dihedral angles for the **D-A** and **D-A-D** fragments were smaller by about eight degrees for the triplet state compared to the singlet state. The effect was even more dramatic in the polymer, with a reduction in dihedral angles of ≤10 degrees. Particularly for the longer polymer fragment with n=7, where the A moiety carrying the maximum spin density is located well within the chain, additionally, a small reduction of the dihedral angles next to those directly adjacent to the dominant A moiety can be observed. While being a rather minor effect, it may well explain the difference in electronic structure of the triplet exciton of **D-A-D** and **(D-A)_n_** evident from the difference in triplet sublevel populations and spectral shape. In the shorter fragment with n=4, such a conclusion would not have been possible due to potential boundary effects, as the dominant A moiety carrying the maximum of spin density is located close to one chain end of the fragment.

We show here only the dihedral angles from geometry optimization performed on the BP86/Def2-SVP level of theory, which is clearly superior over B3LYP/6-31G** for this purpose. Nevertheless, we did perform geometry optimizations for all fragments (excluding the fragment with n=7) using B3LYP/6-31G** as the functional and basis set, respectively. These geometries show the same overall trend in terms of a local planarization due to the triplet state. For details and actual values, see the Supplementary Materials.

For the non-hexylated polymer and its building blocks, no such trend could be deduced due to the dihedral angles between adjacent D and A moieties being always close to zero, in line with an overall pretty flat polymer backbone and a dominating s-shaped curvature.

### 4.5. Different Functionals/Basis Sets for Geometry Optimizations and Spin Density Calculations

In our previous detailed study of the non-hexylated PCDTBT system [39], we did both geometry optimization and spin density calculations on the BP86/Def2-SVP level of theory. This gave consistent results, and already there, we could show BP86/Def2-SVP to be superior over B3LYP/6-31G** for geometry optimization, as judged from consistency with the experimental data. The situation was slightly different for the hexylated compounds, where BP86/Def2-SVP resulted in a spin-density distribution being too delocalized. Nevertheless, the angles from geometry optimization were reasonable. Therefore, we used BP86/Def2-SVP for geometry optimization and performed single-point calculations for the spin-density distribution on the B3LYP/6-31G** level of theory. Note that for calculating magnetic resonance parameters, namely ***D*** tensors, we used EPR-II as the basis set, as it is well known to be suited for those types of calculations.

### 4.6. **D** Tensor Calculations: Challenging But Informative

As is obvious from comparing calculated and experimentally-determined values for |D| and |E| (Table 3), calculating these parameters by using DFT calculations remains challenging. Nevertheless, the calculations revealed details that cannot easily be obtained experimentally, such as the relative orientation of the ***D*** tensor within the molecule (Figure 8) and the sign of the *D* parameter.

**Figure 10:**
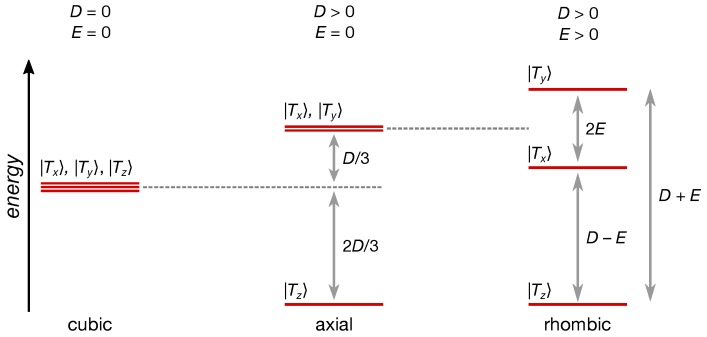
Relative position of the triplet energy sublevels for three characteristic cases. In the case of the compounds investigated here, the situation termed “rhombic” with E≠0 is the relevant one. In this case, all three triplet sublevels can be distinguished by their position in the experimental EPR data. As the three zero-field populations $p_{1,2,3}$ from the simulations are ordered in ascending energy level, they can be assigned to $p_{z,x,y}$, respectively, assuming D>0 as obtained from the DFT calculations.

As mentioned above, the sign of the *D* values calculated using ORCA is always positive. Other studies [77] compared experimentally-obtained signs of *D* with experimental values in more detail, showing excellent agreement between calculations and experiments in this respect. Therefore, we are quite confident that we can assign a positive sign to *D* in our case. Experimental validation would require EPR measurements at low temperatures [79], optically-detected EPR [80,81,82], static magnetic susceptibility measurements [83], or alternatively comparison with other magnetic interaction parameters, preferably hyperfine couplings, if their sign is known [84]. None of these is simply accessible, the latter most probably impossible for the system under investigation, as preliminary ENDOR (electron nuclear double resonance) measurements did not result in any usable signal intensity. Assuming a positive sign for *D* based on the DFT calculations resulted in an oblate spin-density distribution and allowed assigning the populations $p_{1,2,3}$ to triplet energy levels $T_{x,y,z}$ using the conventions given above. The relative arrangement of the three triplet energy levels for D>0 and E/D>0 is given in Figure 10. As the three triplet sublevel populations obtained by spectral simulations were always sorted in ascending order of triplet sublevel energy, we can therefore make the following assignments: $p_{1}\to p_{z}$, $p_{2}\to p_{x}$, and $p_{3}\to p_{y}$. For convenience, a summary of the simulation parameters with these assignments is presented in Table 4.

Based on the assignment of a positive sign to *D* from DFT calculations and given the information of the orientation of the ***D*** tensor within the molecule, we can draw some conclusions from the zero-field triplet sublevel populations obtained by spectral simulations. The vanishing contribution of $p_{z}$ associated with the $D_{z}$ component oriented perpendicular to the aromatic plane, in line with magnetophotoselection experiments on the (non-hexylated) **D-A** fragment [55], can be rationalized based on the spin-density distribution obtained from DFT calculations. However, we would not try to deduce, in reverse, a positive sign of *D* only based on a disk-like (oblate) spin-density distribution of a planar aromatic system.

Having assigned the *x* and *y* axes of the ***D*** tensor based on our convention stated above (Figure 8), we can proceed with a more detailed analysis of the remaining $p_{x}$ and $p_{y}$ populations. As a general trend, the longer the fragment, the larger the population is found along the *x* direction, perpendicular to the backbone. In compound **A**, the dominant Intersystem Crossing (ISC) took place along the *y* axis connecting the two donor thiophenes with the central BT moiety. This is in line with the rather strong donor-acceptor character of the TBT unit [37]. For both **D-A** and **D-A-D**, nearly identical populations $p_{x}$ and $p_{y}$ were revealed, with a clearly higher contribution along the *x* axis, perpendicular to the backbone. This can be rationalized by the curvature introduced by the additional D moieties bearing some spin density, as apparent from the spin-density distribution (Figure 6). Obviously, the additional D moiety in the **D-A-D** fragment only leads to an increased delocalization by extending the conjugated system, but not to an alteration in the orbitals contributing to the ISC. Carefully comparing the TREPR spectra of both compounds shows the only change to be the overall width, hence delocalization. The changes in populations from **A** to **D-A** can be similarly seen in a slight change in spectral shape.

As mentioned already above, proceeding from **D-A-D** to **(D-A)_n_** comes along with a notable change in the overall spectral shape, reflected in the altered triplet sublevel populations $p_{x,y}$, although the overall widths (and hence, the *D* value) of both spectra were identical within experimental error. The increased contribution to the ISC along the molecular *x* direction, perpendicular to the backbone, can be rationalized by the smaller dihedral angles between D and A moieties in **(D-A)_n_** compared to **D-A-D** (Table 2). The triplet exciton seems to flatten the local polymer backbone, most prominently directly adjacent to the central A moiety carrying the maximum spin density, but even extending to the next dihedral angles on each side. This comparably more planar geometry allows for a better conjugation and hence more contribution of the two D moieties, besides increasing the local curvature and thus the contribution to the ISC along the *x* direction.

The detailed discussion of the electronic structure and in particular the origins of the altered zero-field triplet sublevel populations for **D-A-D** and **(D-A)_n_** above has been based on assigning a positive sign to *D* obtained from DFT calculations. However, even without determining or assigning the sign of *D*, the differences in populations observed experimentally reveal them to be a very sensitive probe for the local environment of the triplet exciton. This shows the power of TREPR spectroscopy to reveal even subtle differences in electronic structure and to assign them to a change in (local) geometry of the polymer backbone.

### 4.7. Four Distinct Factors Determining Exciton Delocalization

**Figure 11:**
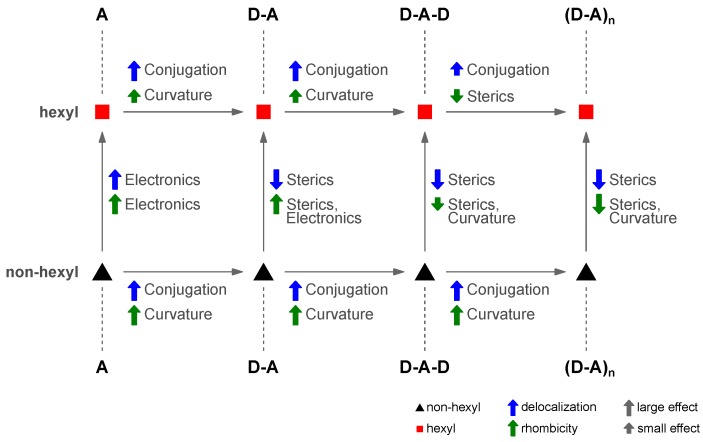
Factors determining the delocalization and triplet exciton rhombicity of the excited states of PCDTBT and its building blocks. Four distinct contributions can be differentiated: electronics (+I effect), the curvature of the polymer backbone, conjugation length, and sterics due to dihedral angles between the D and A moieties.

By comparing both non-hexylated and hexylated polymer and the respective building blocks, four distinct factor determining exciton delocalization and triplet exciton rhombicity can be distinguished (Figure 11): electronics (+I effect), curvature of the polymer backbone, conjugation length, and dihedral angles between D and A moieties due to sterics.

For the non-hexylated system, only increasing conjugation length of the polymer backbone and increasing curvature were relevant and have both a large effect when proceeding from one compound to the next-larger one (cf. Figure 9). In all cases, both, delocalization and rhombicity increased monotonically from smallest to largest fragment investigated. This changes somewhat for the hexylated system. Here, increased conjugation length is still the driving force behind the larger delocalization with enhanced backbone length. However, this effect diminished when proceeding from **D-A-D** to **(D-A)_n_**, with a slightly enhanced delocalization length of the singlet exciton and a near-identical size of the triplet exciton for both molecules. The smaller increase of the rhombicity from **A** to **D-A-D** for the hexylated vs. non-hexylated system can be attributed to the dihedral angles between the D and A moieties in the former, leading to a smaller curvature of the backbone. This effect is slightly reversed for the polymer due to the triplet exciton planarizing locally the polymer backbone, and to a much larger extent than in the smaller fragments. The increased delocalization of the hexylated **A** moiety compared to its non-hexylated counterpart is at first sight surprising. However, it can be ascribed to the +I effect dominating more for the singlet exciton than the triplet exciton. In the case of the triplet exciton for the polymer repeat unit **D-A**, the +I effect gets compensated for by sterical effects of the hexyl side chains resulting in substantial dihedral angles between the D and A moiety. Generally, singlet exciton delocalization seems less affected by dihedral angles and a twisted backbone than triplet excitons. This is in line with previous results on PNDIT2 showing overall more planar geometries of the triplet geometries, but much stronger restricted triplet exciton delocalization as compared to the corresponding singlet excitons [38]. In the symmetric fragment **D-A-D**, finally, sterics reduced the overall curvature in the case of the hexylated compound, resulting in a smaller overall rhombicity of its triplet exciton as compared to the non-hexylated counterpart. The same is true for comparing the two polymers, although here, the effect of reduced impact of the backbone curvature was more pronounced, in line with the smaller dihedral angles found for the polymer compared to **D-A-D**.

Taken together, introducing alkyl side chains, mostly for better solubility and enhanced miscibility with other components, can have a wealth of effects on both the morphology and electronic structure of the underlying polymer that can only be distinguished and investigated in detail by comparing both alkylated and non-alkylated polymers, as well as their respective building blocks of different length.

## 5. Conclusions

In summary, we have investigated the effect of adding alkyl side chains to the electronic structure of the underlying polymer in great detail. For PCDTBT, alkylation generally leads to more localized excitons, but most prominent only for the polymer. Furthermore, singlet excitons seem to be more delocalized than the corresponding triplet excitons, despite the larger dihedral angles between the D and A moieties found for the singlet-state geometries. Using a series of building blocks with increasing length leads to a fundamental understanding of the electronic structure and allows for discriminating different effects, namely electronics (+I effect), curvature, conjugation length, and sterics (backbone twist). Finally, DFT calculation of ***D*** tensors, while still rather limited in terms of the *D* and *E* values obtained, reveals the sign of the *D* value. This allows us to assign the populations to triplet sublevels and to the geometry of the molecule, providing additional insight into the impact even slight modifications of the backbone geometry have on the electronic structure of the excitons. This renders TREPR spectroscopy of triplet excitons well-suited to investigate crucial aspects of the structure-function relationship of conjugated polymers used as organic semiconductors on a molecular basis.

## Figures and Tables

**Table 1:** Simulation parameters for the spectral simulations of the TREPR spectra shown in Figure 4. $\lambda_{\mathrm{ex}}$ is the excitation wavelength used (maximum of the CT band); *D* and *E* are the parameters of the zero-field splitting tensor of the dipolar interaction; $\Gamma_{L}$ is the Lorentzian line width; and $p_{1,2,3}$ are the populations of the three triplet sublevels, respectively, ordered in ascending energy. For details of the fitting procedure, see the Supplementary Materials.

Compound	$\lambda_{\mathrm{ex}}$ (nm)	|D| (MHz)	|E| (MHz)	|E|/|D|	$\Gamma_{L}$ (mT)	$p_{1,2,3}$
**A**	466	1539±2.5	88±1.1	0.057	1.72±0.10	0.000,0.152,0.848
**D-A**	490	1457±2.1	91±0.9	0.062	1.84±0.11	0.000,0.256,0.744
**D-A-D**	509	1387±1.9	96±0.8	0.069	1.38±0.07	0.000,0.264,0.736
**(D-A)_n_**	518	1384±3.1	87±1.4	0.063	2.12±0.15	0.000,0.436,0.564

**Table 2:** Dihedral angles between the aromatic planes of the Cbz and hexTBT moieties obtained from geometry optimization in both the singlet and triplet state. The Cbz and hexTBT moieties are planar. Angles are given in degrees and for the cis-trans configuration as shown in Figure 6. All geometries have been optimized on the BP86/Def2-SVP level of theory. Note that the longer fragment with n=7 has a D moiety on both ends. Bold numbers for the triplet state geometries denote the angles directly adjacent to the A moiety carrying the maximum spin density. The spin density is always centered on one A moiety, cf. Figure 6. For details of how these angles have been obtained, see the Supplementary Materials.

Compound	State	Dihedral Angles													
**D-A**	singlet	35.4													
	triplet	**27.0**													
**D-A-D**	singlet	37.2	35.8												
	triplet	**28.7**	**27.0**												
**(D-A)_4_**	singlet	36.2	31.7	39.3	34.3	35.2	35.1	35.4							
	triplet	36.6	34.3	38.1	30.3	**22.5**	**25.2**	34.0							
**(D-A)_7_-D**	singlet	38.2	37.3	38.7	36.0	41.3	32.5	36.4	32.9	33.4	33.0	38.2	38.3	35.2	34.9
	triplet	39.3	34.7	36.5	31.8	**23.3**	**24.5**	32.4	36.5	43.2	39.3	35.7	34.7	35.5	36.1

**Table 3:** Comparison of calculated and experimental ***D*** tensors, as well as their orientation within the molecular reference frame. ***D*** tensors for each of the compounds have been calculated using the B3LYP/EPR-II level of theory. Values for |D| and |E| are given in MHz. For the orientation of the ***D*** tensor with respect to the molecular reference frame $R_{i}$ with i={x,y,z}, cf. Figure 8. The angles α, β, and γ (in degrees) refer to the deviation of the corresponding ***D*** tensor axes from the molecular reference frame. Only for the asymmetric repeat unit **D-A**, a slight deviation from the molecular reference frame has been obtained, with the *x* and *y* axis tilted towards the additional D moiety. $\rho_{\mathrm{TBT}}$ denotes the relative amount of spin density on the dominating A moiety. Note that for the calculated values, the oligomer fragment with n=4 has been used. The experimental values have been extracted from the simulations shown in Figure 4, cf. Table 1.

Compound	|D|	|E|	|E|/|D|	|D|	|E|	|E|/|D|	α	β	γ	$\rho_{\mathrm{TBT}}$
	Calculated			Experimental						
**A**	808	179	0.22	1539	88	0.06	0.0	0.0	0.0	1.00
**D-A**	736	173	0.24	1457	91	0.06	2.3	2.3	0.2	0.92
**D-A-D**	688	170	0.25	1387	96	0.07	0.1	0.2	0.2	0.88
**(D-A)_n_**	675	173	0.26	1384	87	0.06	0.2	0.0	0.2	0.85

**Table 4:** Simulation parameters for the spectral simulations of the TREPR spectra shown in Figure 4. $\lambda_{\mathrm{ex}}$ is the excitation wavelength used (maximum of the CT band); *D* and *E* are the parameters of the zero-field splitting tensor of the dipolar interaction; $\Gamma_{L}$ is the Lorentzian line width; and $p_{1,2,3}$ are the populations of the three triplet sublevels, respectively, ordered in ascending energy. For actual simulations and details of the fitting procedure, see the Supplementary Materials.

Compound	|D| (MHz)	|E| (MHz)	|E|/|D|	$p_{x}$	$p_{y}$	$p_{z}$
**A**	1539±2.5	88±1.1	0.057	0.152	0.848	0.000
**D-A**	1457±2.1	91±0.9	0.062	0.256	0.744	0.000
**D-A-D**	1387±1.9	96±0.8	0.069	0.264	0.736	0.000
**(D-A)_n_**	1384±3.1	87±1.4	0.063	0.436	0.564	0.000

## Supplementary Materials

The following are available online at https://www.mdpi.com/2073-4360/11/5/870/s1: EPR instrumentation, details of spectral simulations, Figure S1: Characteristics of TREPR spectra of (photo-generated) triplet states, Figure S2: Full 2D TREPR datasets, Figure S3: Temperature-dependent absorption spectra, Figure S4: Comparison of absorption spectra of non-hexylated and hexylated compounds, Figure S5: Comparison of TREPR spectra of non-hexylated and hexylated compounds, Table S1: Simulation parameters for spectral simulations of TREPR spectra, Figure S6: Scheme for the dihedral angles between D and A moieties, Table S2: Dihedral angles for non-hexylated and hexylated compounds, Table S3: Absolute amount of spin density on the TBT moiety for non-hexylated and hexylated compounds, Figure S7: Optimized geometries, Figure S8: Orientation of the calculated ***D*** tensor and molecular reference frame, Table S4: Calculated and experimental ***D*** tensors for non-hexylated and hexylated compounds.

## Abbreviations

The following abbreviations are used in this manuscript:

A	Acceptor
Cbz	Carbazole
COSMO	Conductor-like Screening Model
CT	Charge Transfer
D	Donor
DFT	Density Functional Theory
ENDOR	Electron Nuclear Double Resonance
EPR	Electron Paramagnetic Resonance
ISC	Intersystem Crossing
PCDTBT	Poly[*N*-9${}^{\prime }$-heptadecanyl-2,7-carbazole-*alt*-5,5-(4${}^{\prime }$,7${}^{\prime }$-di-2-thienyl-2${}^{\prime }$,1${}^{\prime }$,3${}^{\prime }$-benzothiadiazole)]
PNDIT2	Poly{[*N*,*N*${}^{\prime }$-bis(2-octyldodecyl)-naphthalene-1,4,5,8-bis(dicarboximide)-2,6-diyl]-*alt*-5,5${}^{\prime }$-(2,2${}^{\prime }$-bithiophene)}
TBT	Dithienyl-Benzothiadiazole
TREPR	Time-Resolved Electron Paramagnetic Resonance
ZFS	Zero-Field Splitting
