# Supplementary material for: Impact of Side Chains of Conjugated Polymers on Electronic Structure: A Case Study
Source: Polymers (Basel). 2019 May 13;11(5):870. doi: 10.3390/polym11050870 (PMC6572471; doi:10.3390/polym11050870)
Supplement: Supplementary file 1 [file polymers-11-00870-s001.pdf]

# Supplementary Materials: Side Chain Engineering of PCDTBT: Impact on Electronic Structure

**Clemens Matt [1], Florian Lombeck [2], Michael Sommer [2,†], and Till Biskup [1,*]**

## Contents

1 EPR Instrumentation 1
2 TREPR spectra of triplet states 2
3 Spectral simulations of TREPR spectra of triplet states 2
4 TREPR signal decay and triplet lifetime 5
5 Temperature-dependent absorption spectra 6
6 Comparison of non-hexylated and hexylated compounds 7
7 Determining the angles between TBT and Cbz moieties 9
8 Quantitative analysis of spin density distributions 11
9 Comparison of calculations with BP86 and B3LYP 13
10 *D* tensor calculation 14
References 16

## 1. EPR Instrumentation

TREPR spectroscopy with a time resolution of up to 10 ns allows for real-time observation, *e.g.*, of short-lived radical-pair and triplet states generated by pulsed laser excitation. In contrast to conventional continuous-wave EPR spectroscopy, which usually involves magnetic-field modulation to improve the signal-to-noise ratio, TREPR is recorded in a high-bandwidth direct-detection mode, so as not to constrain the time resolution of the experiment. Consequently, positive and negative signal amplitudes in TREPR correspond to enhanced absorptive (A) and emissive (E) electron-spin polarisations of the EPR transitions, respectively.

All TREPR experiments were performed at 80 K using a commercial EPR spectrometer (Bruker ESP380E) in conjunction with a Bruker microwave bridge (ER 046 MRT) equipped with a low-noise high-bandwidth video amplifier. The sample was placed in a synthetic-quartz (Suprasil) sample tube (3 mm inner diameter) and irradiated in a dielectric-ring resonator (Bruker ER 4118X-MD5), which was immersed in a helium gas-flow cryostat (Oxford CF-935) cooled with liquid nitrogen. The temperature was regulated to ±0.1 K by a temperature controller (Oxford ITC-503). The time resolution of the experimental setup was in the 10 ns range. A microwave frequency counter (Hewlett-Packard HP 5352B) was used to monitor the microwave frequency.

Optical excitation at the respective wavelengths was carried out with an optical parametric oscillator (OPO) system (Opta BBO-355-vis/IR) pumped by an Nd:YAG laser (Spectra Physics, Quanta Ray GCR 190-10) with a pulse width of approximately 6 ns, and a pulse energy of 1 mJ. The repetition rate of the laser was set to 10 Hz. A transient recorder (LeCroy 9354A) with a digitizing rate of 2 ns/11 bit was used to acquire the time-dependent EPR signal. To eliminate the background signal induced by the laser entering the EPR cavity, TREPR signals were accumulated at off-resonance magnetic-field positions (background) and subtracted from those recorded on-resonance. This background signal is

completely independent in its shape from both, laser wavelength and magnetic field, and normally long-lived compared to the detected spin-polarised EPR signal. Background subtraction was performed directly in the transient recorder and a background signal repeatedly recorded after each tenth time trace of the experimental data.

Further experimental parameters (except where explicity given) are as follows: Microwave frequency, 9.700 GHz, microwave power: 2 mW (20 dB attenuation, source power 200 mW), frequency-mixer detection, video amplifier set to 42 dB amplification and 25 MHz bandwidth, between 850 and 1400 averages per point.

## 2. TREPR spectra of triplet states

As TREPR spectra of spin-polarised triplet states of organic molecules recorded at X-band frequencies and magnetic fields are normally dominated by the zero-field splitting (ZFS) interaction, the hamilton operator used to describe the system reduces dramatically. The only contributions that need to be taken into account are the Hamilton operator for the Zeeman interaction, $\mathcal{H}_{\mathrm{EZ}}$, and the one for the ZFS interaction, $\mathcal{H}_{\mathrm{ZFS}}$:

$$\hat{\mathcal{H}} = \hat{\mathcal{H}}_{\mathrm{EZ}} + \hat{\mathcal{H}}_{\mathrm{ZFS}} = g_e \beta_e \boldsymbol{B}^{\mathrm{T}} \cdot \hat{\boldsymbol{S}} + \hat{\boldsymbol{S}}^{\mathrm{T}} \cdot \boldsymbol{D} \cdot \hat{\boldsymbol{S}} \,. \tag{1}$$

All other contributions can be considered as small perturbations that can be accounted for using (inhomogeneous) line broadening.

The $\boldsymbol{D}$ tensor in its principal axis system is given to

$$\boldsymbol{D} = \begin{pmatrix} -\frac{1}{3}D + E & 0 & 0 \\ 0 & -\frac{1}{3}D - E & 0 \\ 0 & 0 & \frac{2}{3}D \end{pmatrix} \tag{2}$$

where $D$ and $E$ are the zero-field-splitting parameters that can be directly read out from the experimental spectra (cf. Fig. S1). Note that $D$ and $E$ are defined such in the simulation routine used that the relation $|E| \leq |D|/3$ always holds.

## 3. Spectral simulations of TREPR spectra of triplet states

All simulations of triplet spectra have been performed using the EasySpin software package [1] available for MATLAB® (MathWorks), and here the routine `pepper`. Parameters included were the $\boldsymbol{g}$ and $\boldsymbol{D}$ tensor and the triplet sublevel populations (in zero field). Line broadening ($\Gamma$) was included using a combination of Lorentzian ($\Gamma_{\mathrm{L}}$) and Gaussian ($\Gamma_{\mathrm{G}}$) lines. For all simulations, the $\boldsymbol{g}$ tensor was assumed to be isotropic, with $g_{\mathrm{iso}} = 2.002$. This left the parameters $D$ and $E$ of the zero-field splitting tensor $\boldsymbol{D}$, the populations $p_1$, $p_2$, and $p_3$, and the two line widths $\Gamma_{\mathrm{L}}$ and $\Gamma_{\mathrm{G}}$ as the only free parameters that were adjusted.

Fitting the spectral simulations to the experimental data was done with the routine `lsqcurvefit` from the MATLAB® Optimization Toolbox™ using the trust-region-reflective least squares algorithm.

The nonlinear least-square solver finds the $m$ coefficients $\boldsymbol{a}$ that solve the problem

$$\min_{\boldsymbol{a}} \sum_i \left(f(x_i; \boldsymbol{a}) - y_i\right)^2 \tag{3}$$

with $y_i$ being the measured data and $f(x_i; \boldsymbol{a})$ the fitting function $f : \mathbb{R}^m \to \mathbb{R}^n$ with the same size $n$ as the measured data $y_i$

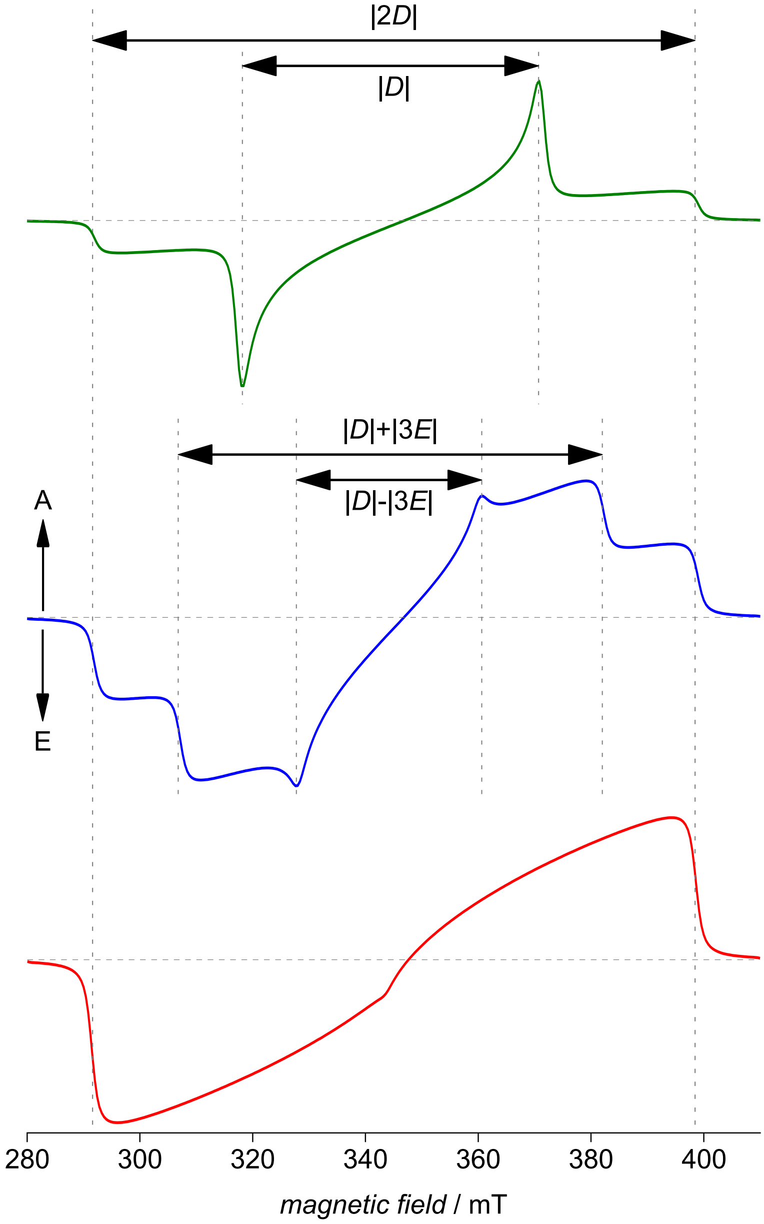


**Figure S1. Characteristics of TREPR spectra of (photo-generated) triplet states.** Three characteristic situations for the ratio of the two parameters $D$ and $E$ of the ZFS tensor are depicted here: the fully axial case (top, green), an intermediate case (blue, centre) and a fully rhombic case (red, bottom). Spectra were calculated using EasySpin. [2] The zero-field populations $p_{1,2,3}$ of the three triplet sublevels are far from thermal equilibrium, due to optical excitation and the inherent anisotropy of the intersystem crossing processes. Therefore, signals consist of both, absorptive (A) and emissive (E) contributions.

Error estimation of the fitting parameters was carried out by using the Jacobian matrix **J**. $J_{ij}$ is the partial derivative of the fitting function $f(x_i; \boldsymbol{a})$ with respect to $a_j$ at the solution $a_0$.

$$J_{ij}(\boldsymbol{a}_0) := \left( \frac{\partial f(x_i; \boldsymbol{a})}{\partial a_j}(\boldsymbol{a}_0) \right)_{i=1...n, j=1...m} \tag{4}$$

$$\mathbf{J}(\boldsymbol{a}_0) = \begin{pmatrix} \frac{\partial f(x_1;\boldsymbol{a})}{\partial a_1}(\boldsymbol{a}_0) & ... & \frac{\partial f(x_1;\boldsymbol{a})}{\partial a_m}(\boldsymbol{a}_0) \\ ... & & \\ \frac{\partial f(x_n;\boldsymbol{a})}{\partial a_1}(\boldsymbol{a}_0) & ... & \frac{\partial f(x_n;\boldsymbol{a})}{\partial a_m}(\boldsymbol{a}_0) \end{pmatrix} \tag{5}$$

The variances of the coefficients $a_j$ are given by the diagonal elements of the covariance matrix, **C**, i.e. $\sigma^2_{a_j} = C_{jj}$, where **C** is the inverse of the matrix **H**, variously referred to as the curvature or Hessian matrix.

The Hessian matrix was approximated by a series expansion, which is terminated after the first rank:

$$H_{jk} = \frac{1}{2} \frac{\partial^2 \chi^2(\boldsymbol{a})}{\partial a_j \partial a_k} \approx \sum_{i=1}^{n} \frac{1}{\sigma_i^2} \frac{\partial f(x_i; \boldsymbol{a})}{\partial a_j} \frac{\partial f(x_i; \boldsymbol{a})}{\partial a_k}$$

Hence the Jacobian matrix can be used to approximate the Hessian if $\sigma_i^2$ is chosen to be equal for all points,

$$\mathbf{H} \approx \frac{1}{\sigma_i^2} \mathbf{J}^{\mathrm{T}} \cdot \mathbf{J}. \tag{6}$$

To speed up calculation time for the matrix product $\mathbf{J}^{\mathrm{T}} \cdot \mathbf{J}$, an economy-size QR decomposition of **J** was carried out, reducing the dimension of **R** to the size of $\boldsymbol{a}$:

$$\mathbf{J} = \mathbf{Q} \cdot \mathbf{R}. \tag{7}$$

In the following matrix multiplication, **Q** vanishes by multiplication with $\mathbf{Q}^{\mathrm{T}}$:

$$(\mathbf{J}^{\mathrm{T}} \cdot \mathbf{J})^{-1} = (\mathbf{R}^{\mathrm{T}} \cdot \mathbf{R})^{-1} = \mathbf{R}^{-1} \cdot (\mathbf{R}^{\mathrm{T}})^{-1} = \mathbf{R}^{-1} \cdot (\mathbf{R}^{-1})^{\mathrm{T}} \tag{8}$$

In MATLAB®, this implementation leads to high computational speed and only minor numerical errors. The corresponding code would be as follows:

```
[~,R] = qr(jacobian,0);
```

The diagonal elements of the approximated $\mathbf{H}^{-1}$ can easily be calculated by element-wise squaring followed by summation over the rows of **R**. Since $\sigma_i^2$ is chosen to be equal for all points, the errors for the fit parameters are given by:

```
stdDev = sqrt(variance * sum(inv(R).^2,2));
```

The fitting algorithm `lsqcurvefit` can optionally return the residuals as additional output argument, here termed `residuals`. Hence the variance of the residuals obtained as

```
variance = var(residuals);
```

was used as $\sigma^2$ for all points.

## 4. TREPR signal decay and triplet lifetime

The kinetics of TREPR signals of triplet states are rather complicated, involving spin relaxation, decay of spin polarisation, and decay of the actual triplet state, usually via intersystem crossing back into the singlet ground state. Hence, only a lower limit of the triplet lifetime can be extracted from the TREPR time profiles, based on the simple fact that regardless of all other processes, TREPR signals will only be observable as long as there exists a triplet state.

As can be seen from the full 2D datasets of all four compounds investigated in this study (Fig. S2), TREPR signals decay on the time scale of several microseconds. However, it is highly likely that this decay is dominated by the microwave power and other experimental parameters and that the actual triplet state lifetime extends well into tens or even hundreds of microseconds.

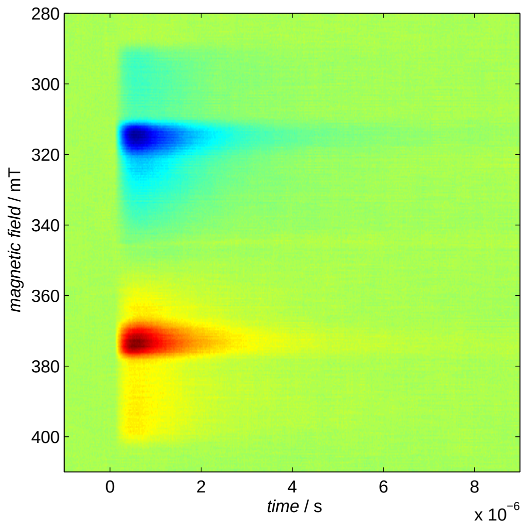


(a) **A** (hexTBT)

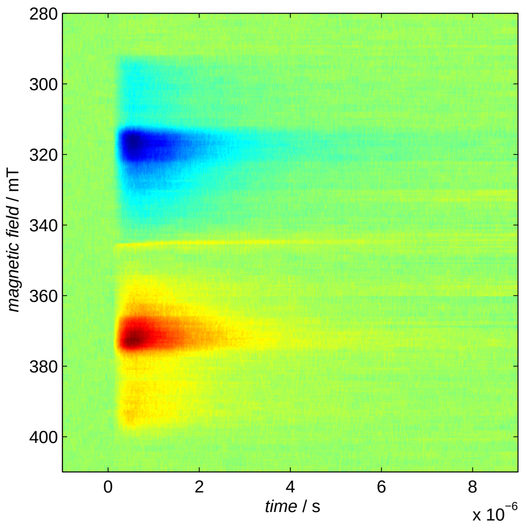


(b) **D-A** (CbzhexTBT)

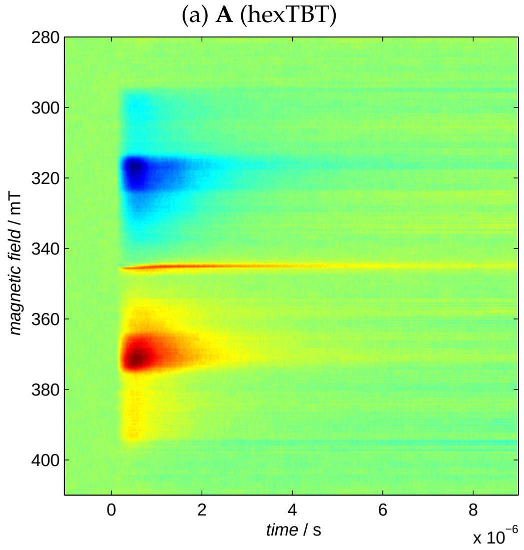


(c) **D-A-D** (CbzhexTBTCbz)

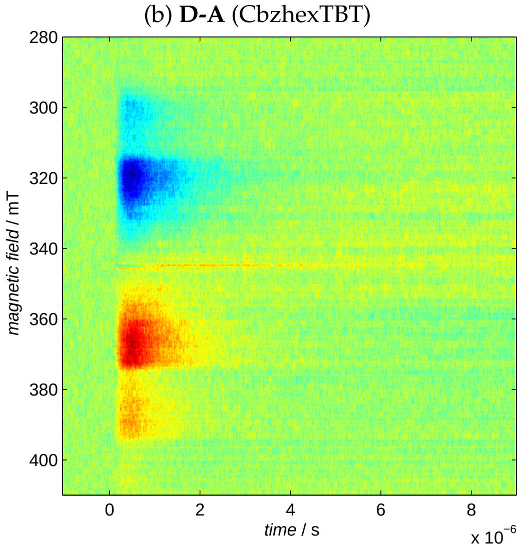


(d) $\mathbf{(D\text{-}A)_n}$ (hexPCDTBT)

**Figure S2. Full 2D TREPR datasets of all compounds investigated in this study.** Blue colour denotes emissive, red colour enhanced absorptive polarisation. For experimental details see main text.

## 5. Temperature-dependent absorption spectra

To rule out effects of aggregation, temperature-dependent absorption spectra have been recorded for all substances investigated. The results are shown in Fig. S3. Due to the solvent, *o*-dichlorobenzene, not forming a transparent glass upon freezing, spectra could only be recorded until the solution froze out. As obvious from the spectra, none of the substances investigated showed any sign of aggregation under the conditions used here.

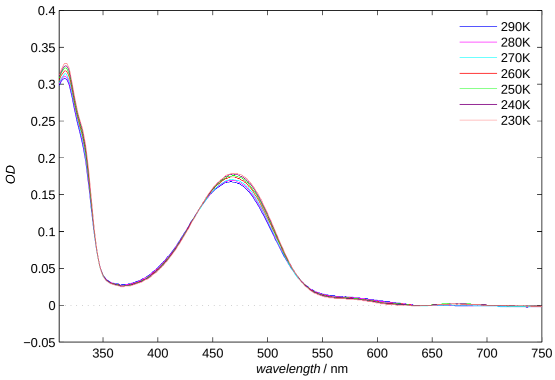


(a) **A** (hexTBT)

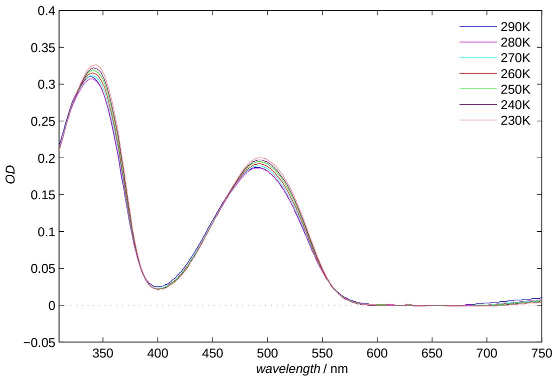


(b) **D-A** (CbzhexTBT)

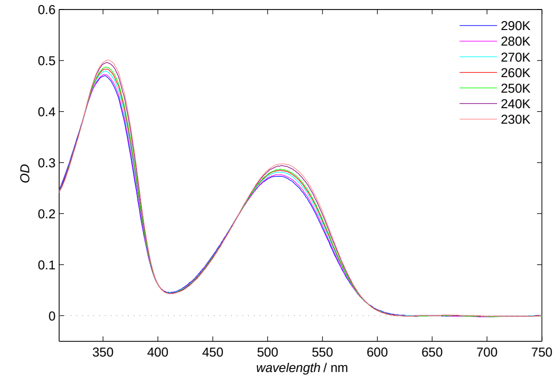


(c) **D-A-D** (CbzhexTBTCbz)

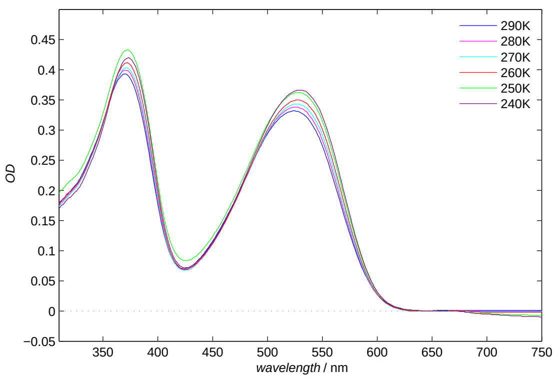


(d) **(D-A)$_{\mathbf{n}}$** (hexPCDTBT)

**Figure S3. Temperature-dependent absorption spectra of all compounds investigated in this study.** Due to the solvent, *o*-dichlorobenzene, not forming a transparent glass upon freezing, spectra could only be recorded until the solution froze out.

## 6. Comparison of non-hexylated and hexylated compounds

The non-hexylated polymer and its building blocks have been investigated previously [2]. For a detailed discussion of the differences and commonalities between the two systems, hexylated and non-hexylated, see the main text. Here, both steady-state absorption spectra (Fig. S4) as well as TREPR data (Fig. S5), are presented face to face for ease of comparison. Additionally, Tab. S1 summarises the simulation parameters for the spectral simulations of both systems.

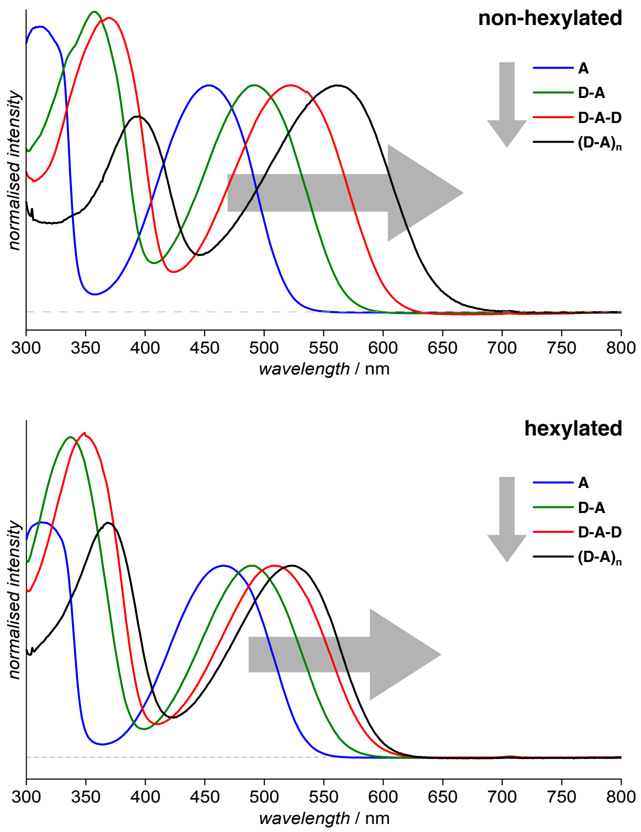


**Figure S4. Comparison of the steady-state absorption spectra of the non-hexylated and hexylated compounds.** All spectra have been recorded at room temperature in standard path length cuvettes in *o*-dichlorobenzene. Data for the non-hexylated compounds (top) taken from Ref. [2].

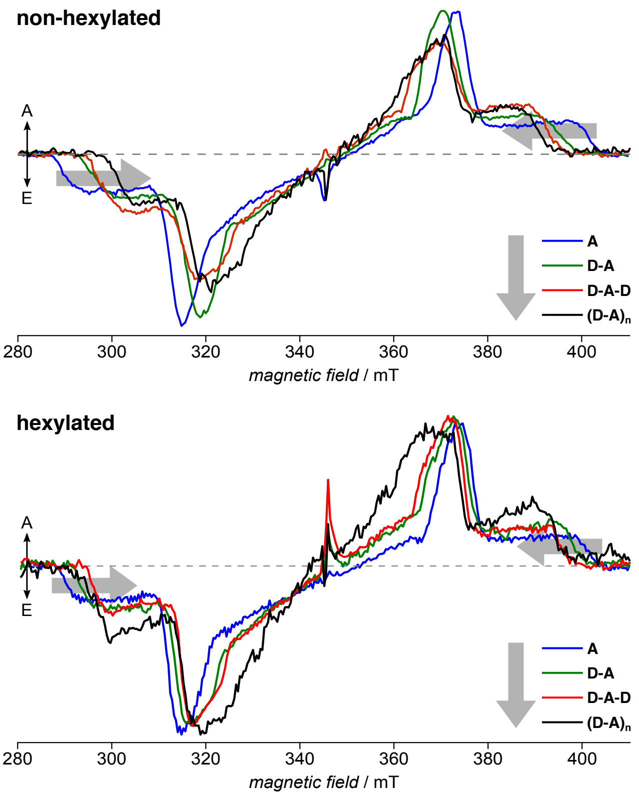


**Figure S5. Comparison of the TREPR spectra of the non-hexylated and hexylated compounds.** All spectra have been recorded at 80 K with the identical setup described above. Samples have been excited with wavelengths corresponding to the maximum of their respective CT band. Data for the non-hexylated compounds (top) taken from Ref. [2].

**Table S1. Simulation parameters for the spectral simulations of the TREPR spectra shown in Fig. S5.** $\lambda_{ex}$ is the excitation wavelength used (maximum of the CT band), $D$ and $E$ are the parameters of the zero-field splitting tensor of the dipolar interaction, $\Gamma_L$ is the Lorentzian line width, and $p_{1,2,3}$ are the populations of the three triplet sublevels, respectively. For actual simulations see the main text and Ref. [2], for details of the fitting procedure see above.

| | $\lambda_{ex}$/nm | $\|D\|$/MHz | $\|E\|$/MHz | $\|E\|/\|D\|$ | $\Gamma_L$/mT | $p_{1,2,3}$ |
|---|---|---|---|---|---|---|
| | | | non-hexylated | | | |
| **A** | 454 | 1550 ± 2.2 | 70 ± 1.0 | 0.045 | 2.36 ± 0.09 | 0.000, 0.182, 0.818 |
| **D-A** | 492 | 1362 ± 2.5 | 76 ± 1.4 | 0.056 | 3.42 ± 0.13 | 0.000, 0.138, 0.862 |
| **D-A-D** | 522 | 1303 ± 3.6 | 101 ± 1.7 | 0.078 | 2.96 ± 0.16 | 0.000, 0.309, 0.691 |
| **$(D\text{-}A)_n$** | 550 | 1254 ± 3.0 | 101 ± 1.4 | 0.081 | 2.39 ± 0.13 | 0.000, 0.351, 0.649 |
| | | | hexylated | | | |
| **A** | 466 | 1539 ± 2.5 | 88 ± 1.1 | 0.057 | 1.72 ± 0.10 | 0.000, 0.152, 0.848 |
| **D-A** | 490 | 1457 ± 2.1 | 91 ± 0.9 | 0.062 | 1.84 ± 0.11 | 0.000, 0.256, 0.744 |
| **D-A-D** | 509 | 1387 ± 1.9 | 96 ± 0.8 | 0.069 | 1.38 ± 0.07 | 0.000, 0.264, 0.736 |
| **$(D\text{-}A)_n$** | 518 | 1384 ± 3.1 | 87 ± 1.4 | 0.063 | 2.12 ± 0.15 | 0.000, 0.436, 0.564 |

## 7. Determining the angles between TBT and Cbz moieties

As the aromatic planes of the TBT and Cbz moieties can be twisted against each other in all three directions, the tilting cannot be ascribed fully by single dihedral angle between adjacent atoms. Hence, coordinate systems have been created in both aromatic planes and the angles between the $x$, $y$, and $z$ axes compared to those of the planar structure.

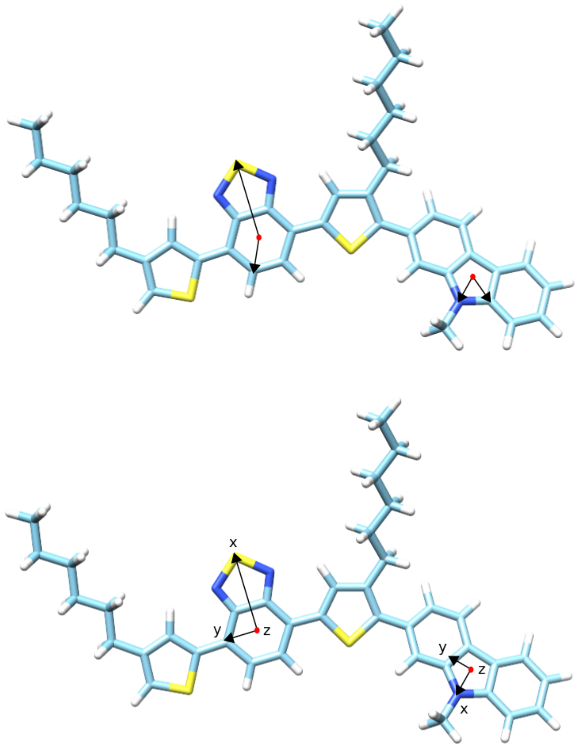


**Figure S6. Illustration of constructing the vectors that have been used for determining the angles between TBT and Cbz moieties.** The top panel shows the two vectors spanning the aromatic planes, whereas the complete coordinate systems in Cbz and TBT are shown in the bottom panel.

In a first step, the center of both, benzene and pyrroline rings have been calculated (red dot in Fig. S6) and the vectors from there to the S and N atom, respectively, created that form the $x$ axis of the coordinate system. A second vector has been created pointing from the centre of the aromatic plane to one of the atoms in the aromatic plane to span this plane (Fig. S6, top). The cross product of these two vectors led to the $z$ axis of the coordinate system. In the following, the cross product of the vectors for the $x$ and $z$ axis could be used to create the $y$ axis and hence complete the coordinate system (Fig. S6, bottom).

As the cross product always generates one of two possible vectors being orthogonal to the two vectors multiplied, displaying all vectors was necessary to check that the correct vector has been found. If not, the two vectors for the cross product have to be swapped.

For convenience, the angles for the cis-trans configuration given already in the main text are shown in Tab. S2 for both types of calculations performed, with BP86/Def2-SVP and B3LYP/6-31G**. As can be readily seen, geometries optimised using BP86/Def2-SVP are very flat, whereas particularly for the polymer, B3LYP/6-31G** predicts a twist in the backbone that seems not to be in line with our experimental results. For a detailed discussion, the reader is referred to the main text.

**Table S2. Dihedral angles between the aromatic planes of Cbz and hexTBT moieties obtained from geometry optimisation in both, singlet and triplet state.** The Cbz and hexTBT moieties are in themselves pretty flat. Angles are given in degrees and for the cis-trans configuration. Note that the longer fragment with $n = 7$ has a D moiety on both ends. Bold numbers for the triplet state geometries denote the angles directly adjacent to the A moiety carrying the maximum spin density. The spin density is always centred on one A moiety.

| compound | state | dihedral angles | | | | | | | | | | | | | |
|---|---|---|---|---|---|---|---|---|---|---|---|---|---|---|---|
| | | non-hexylated, BP86/Def2-SVP | | | | | | | | | | | | | |
| **D-A** | singlet | 3.0 | | | | | | | | | | | | | |
| | triplet | **1.1** | | | | | | | | | | | | | |
| **D-A-D** | singlet | 1.0 | 3.2 | | | | | | | | | | | | |
| | triplet | **0.7** | **0.4** | | | | | | | | | | | | |
| **(D-A)$_4$** | singlet | 0.9 | 0.6 | 1.6 | 3.9 | 3.1 | 3.5 | 1.4 | | | | | | | |
| | triplet | 1.0 | 0.2 | **0.5** | **1.1** | 1.4 | 2.9 | 1.0 | | | | | | | |
| | | non-hexylated, B3LYP/6-31G** | | | | | | | | | | | | | |
| **D-A** | singlet | 16.6 | | | | | | | | | | | | | |
| | triplet | **7.2** | | | | | | | | | | | | | |
| **D-A-D** | singlet | 16.7 | 16.7 | | | | | | | | | | | | |
| | triplet | **0.8** | **5.9** | | | | | | | | | | | | |
| **(D-A)$_4$** | singlet | 14.2 | 14.5 | 20.4 | 17.0 | 20.0 | 12.2 | 14.2 | | | | | | | |
| | triplet | 17.2 | 12.2 | **10.1** | **9.0** | 11.7 | 19.8 | 13.8 | | | | | | | |
| | | hexylated, BP86/Def2-SVP | | | | | | | | | | | | | |
| **D-A** | singlet | 35.4 | | | | | | | | | | | | | |
| | triplet | **27.0** | | | | | | | | | | | | | |
| **D-A-D** | singlet | 37.2 | 35.8 | | | | | | | | | | | | |
| | triplet | **28.7** | **27.0** | | | | | | | | | | | | |
| **(D-A)$_4$** | singlet | 36.2 | 31.7 | 39.3 | 34.3 | 35.2 | 35.1 | 35.4 | | | | | | | |
| | triplet | 36.6 | 34.3 | 38.1 | 30.3 | **22.5** | **25.2** | 34.0 | | | | | | | |
| **(D-A)$_7$-D** | singlet | 38.2 | 37.3 | 38.7 | 36.0 | 41.3 | 32.5 | 36.4 | 32.9 | 33.4 | 33.0 | 38.2 | 38.3 | 35.2 | 34.9 |
| | triplet | 39.3 | 34.7 | 36.5 | 31.8 | **23.3** | **24.5** | 32.4 | 36.5 | 43.2 | 39.3 | 35.7 | 34.7 | 35.5 | 36.1 |
| | | hexylated, B3LYP/6-31G** | | | | | | | | | | | | | |
| **D-A** | singlet | 38.4 | | | | | | | | | | | | | |
| | triplet | **34.4** | | | | | | | | | | | | | |
| **D-A-D** | singlet | 40.8 | 40.1 | | | | | | | | | | | | |
| | triplet | **36.1** | **36.0** | | | | | | | | | | | | |
| **(D-A)$_4$** | singlet | 43.5 | 35.6 | 39.0 | 37.3 | 46.0 | 39.9 | 32.5 | | | | | | | |
| | triplet | 38.3 | 38.0 | **35.7** | **36.3** | 39.7 | 39.2 | 33.3 | | | | | | | |

## 8. Quantitative analysis of spin density distributions

Whereas spin density plots on the optimised geometries of the molecules are very valuable to give an overall impression, they are less useful for a quantitative comparison.

ORCA normalises the calculated spin densities internally such that the sum of all spin densities equals to the number of spins involved. In case of a triplet state with two unpaired electron spins, this sum equals to two.

To calculate the relative amount of spin density on the TBT moiety, $r^{\text{abs}}_{\text{TBT}}(\text{M})$, for all compounds, M, investigated, the spin density on the TBT moiety, $\rho_{\text{TBT}}(\text{M})$, has been divided by the spin density on the entire molecule, $\rho_{\text{M}}(\text{M})$ as follows:

$$r^{\text{abs}}_{\text{TBT}}(\text{M}) = \frac{\sum |\rho_{\text{TBT}}(\text{M})|}{\sum |\rho_{\text{M}}(\text{M})|} \leq 1\,.$$

As spin densities can have positive and negative sign, the absolute values have been taken in this case, hence the notation $r^{\text{abs}}$. The resulting values can be found in Tab. S3 and graphically displayed in Fig. S7.

**Table S3. Absolute amount of spin density on the TBT moiety as calculated with BP86 and B3LYP functionals on both geometries.** Geometries have been optimised on the BP86/Def2-SVP and B3LYP/6-31G** level of theory, and spin densities calculated for both geometries and both functional/basis set combinations.

| Geometry | Spin Density | **A** | **D-A** | **D-A-D** | **(D-A)$_\text{n}$** |
|---|---|---|---|---|---|
| | | non-hexyl | | | |
| BP86/Def2-SVP | BP86/Def2-SVP | 1.00 | 0.87 | 0.77 | 0.52 |
| BP86/Def2-SVP | B3LYP/6–31G** | 1.00 | 0.89 | 0.84 | 0.80 |
| B3LYP/6–31G** | BP86/Def2-SVP | 1.00 | 0.88 | 0.78 | 0.71 |
| B3LYP/6–31G** | B3LYP/6–31G** | 1.00 | 0.90 | 0.85 | 0.84 |
| | | hexyl | | | |
| BP86/Def2-SVP | BP86/Def2-SVP | 1.00 | 0.91 | 0.86 | 0.72 |
| BP86/Def2-SVP | B3LYP/6–31G** | 1.00 | 0.92 | 0.88 | 0.85 |
| B3LYP/6–31G** | BP86/Def2-SVP | 1.00 | 0.92 | 0.86 | 0.86 |
| B3LYP/6–31G** | B3LYP/6–31G** | 1.00 | 0.94 | 0.90 | 0.91 |

To get further insight, spin densities have been compared for the TBT unit in each of the compounds in form of a histogram. Therefore, spin densities of the TBT unit have to be normalised to the amount of spin density on the TBT moiety in the TBT compound, $\rho_{\text{TBT}}(\text{TBT})$, relative to the amount of spin density on the TBT moiety in the actual compound, $\rho_{\text{TBT}}(\text{M})$, and this time, the sign of the spin densities has to be taken into account:

$$r_{\text{TBT}}(\text{M}) = \frac{\sum \rho_{\text{TBT}}(\text{M})}{\sum \rho_{\text{TBT}}(\text{TBT})} \leq 1\,.$$

Note that, in our case, $\sum \rho_{\text{TBT}}(\text{TBT})$ does not amount to two, as one would naively expect, as the two protons present in TBT that are substituted successively in the larger compounds are not taken into account for calculating the ratio of the spin densities, but do possess some non-vanishing, although rather small, spin density. Hence this rather complicated way of calculating this ratio.

Having the two ratios of spin density on the TBT moiety as compared to the entire molecule at hand, we can proceed to calculate the correction factor $r_{\mathrm{corr}}(\mathrm{M})$ for the spin density values for the TBT moiety of a given compound:

$$r_{\mathrm{corr}}(\mathrm{M}) = \frac{r_{\mathrm{TBT}}^{\mathrm{abs}}(\mathrm{M})}{r_{\mathrm{TBT}}(\mathrm{M})}$$

and with this, the corrected spin density for the TBT moiety of a given compound amounts to:

$$\rho_{\mathrm{TBT}}^{\mathrm{corr}}(\mathrm{M}) = r_{\mathrm{corr}}(\mathrm{M}) \cdot \rho_{\mathrm{TBT}}(\mathrm{M})\,.$$

The values for $\rho_{\mathrm{TBT}}^{\mathrm{corr}}(\mathrm{M})$ for each of the atoms have been displayed in the respective histograms.

## 9. Comparison of calculations with BP86 and B3LYP

As mentioned in the main text, geometry optimisations and spin density calculations of all compounds investigated have been performed both, with BP86/Def2-SVP [3–5] and B3LYP/6–31G** [6–9] as functional and basis set, respectively, and the results compared with each other. For all calculations, ORCA 3.0.3 [10] has been used. The solvent has been accounted for by the COSMO model [11]. Initial geometries of the molecules were created using Avogadro 1.1.1 [12]. Spin density plots were created using UCSF Chimera 1.11.2 [13].

For all four compounds investigated, geometry optimisations of their respective triplet states have been performed. The two sets of calculations differ clearly in the degree of planarity of the resulting geometries, as can be readily seen from a side view parallel to the aromatic planes (Fig. S7). For actual numbers of the dihedral angles between adjacent aromatic planes of Cbz and TBT moieties, cf. Tab. S2.

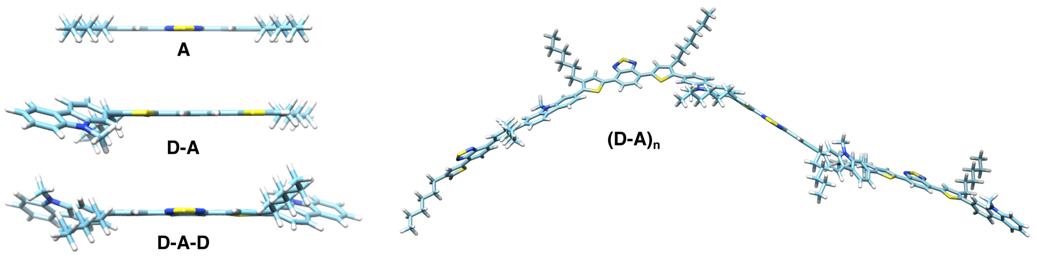


(a) BP86/Def2-SVP

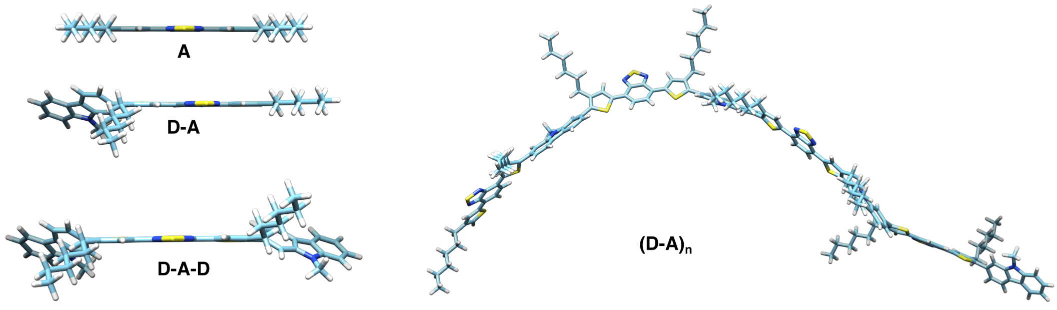


(b) B3LYP/6-31G**

**Figure S7. Side-view of the optimised geometries of the triplet states of PCDTBT and its building blocks.** Geometries have been optimised for the triplet state on the theory level BP86/Def2-SVP and B3LYP/6–31G**, respectively. As obvious from this side-view, all molecules are rather flat for BP86/Def2-SVP, with dihedral angles between the aromatic planes of Cbz and TBT, respectively, close to zero, whereas B3LYP/6–31G** results in clear distortions. For actual values of these dihedral angles, cf. Tab. S2.

Whereas the geometries calculated using BP86/Def2-SVP are basically flat, for B3LYP/6–31G**, particularly the polymer fragment with $n = 4$ shows clear distortions with dihedral angles up to approx. 20%.

## 10. *D* tensor calculation

We calculated the ***D*** tensor for each of the compounds for both geometries (using BP86/Def2-SVP and B3LYP/6-31G** level of theory, respectively) using B3LYP as functional and EPR-II [14] as basis set. Interestingly, all calculated tensors exhibit a mutually identical orientation within the molecular frame, with their $x$ and $y$ axes within the aromatic plane of the acceptor moiety and the $z$ axis perpendicular to it. The $x$ axis points perpendicular to the axis connecting thiophene–benzothiadiazole and the $y$ axis along this connection (Figure S8). Assigning the ***D*** tensor axes is based on the usual convention $|D_z| > |D_y| > |D_x|$, assuming, *inter alia*, $|E| \leq |D|/3$ [15] and $E/D > 0$.

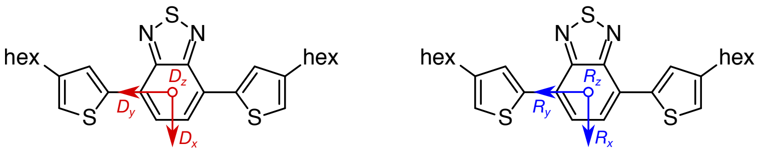


**Figure S8. Orientation of the calculated *D* tensor within the TBT acceptor moiety and molecular reference frame.** For each of the fragments investigated, the ***D*** tensor is basically oriented in the same way, as shown on the left. Assuming a right-handed coordinate system, the $z$ component is pointing towards the paper plane. Only for the asymmetric repeat unit **D-A** a slight deviation from the molecular reference frame ($R_i$ with $i = \{x, y, z\}$, right) of a few degrees has been obtained from the DFT calculations. The deviation from the reference frame is given as three dihedral angles, $\alpha$, $\beta$, and $\gamma$, for each of the three axes, $x$, $y$, and $z$, respectively. For actual values of these angles see Table S4.

The molecular reference frame is given in Figure S8 (right), with the $x$ and $y$ axes as well within the aromatic plane of the acceptor moiety and the $z$ axis perpendicular to it, accordingly. The deviations (dihedral angles) of the ***D*** tensor axes from the molecular reference frame are given in Table S4.

**Table S4. Comparison of calculated and experimental *D* tensors as well as their orientation within the molecular reference frame.** ***D*** tensors for each of the compounds have been calculated using the B3LYP/EPR-II level of theory. Values for $|D|$ and $|E|$ are given in MHz. For the orientation of the ***D*** tensor with respect to the molecular reference frame $R_i$ with $i = \{x, y, z\}$ cf. Figure S8. The angles $\alpha$, $\beta$, and $\gamma$ (in degrees) refer to the deviation of the corresponding ***D*** tensor axes from the molecular reference frame. Only for the asymmetric repeat unit **D-A**, a slight deviation from the molecular reference frame has been obtained, with the $x$ and $y$ axis tilted towards the additional D moiety. Note that for the calculated values, the oligomer fragment with $n = 4$ has been used.

| compound | $\|D\|$ | $\|E\|$ calculated | $\|E\|/\|D\|$ | $\|D\|$ | $\|E\|$ experimental | $\|E\|/\|D\|$ | $\alpha$ | $\beta$ | $\gamma$ |
|---|---|---|---|---|---|---|---|---|---|
| non-hexylated, geometry: BP86/Def2-SVP | | | | | | | | | |
| **A** | 847 | 179 | 0.21 | 1550 | 70 | 0.06 | 0.0 | 0.0 | 0.0 |
| **D-A** | 730 | 180 | 0.25 | 1362 | 76 | 0.06 | 4.0 | 4.1 | 0.5 |
| **D-A-D** | 662 | 174 | 0.26 | 1303 | 101 | 0.07 | 0.1 | 0.2 | 0.2 |
| **(D-A)$_n$** | 662 | 180 | 0.27 | 1254 | 101 | 0.06 | 0.0 | 0.1 | 0.1 |
| non-hexylated, geometry: B3LYP/6-31G** | | | | | | | | | |
| **A** | 857 | 183 | 0.21 | 1550 | 70 | 0.06 | 0.0 | 0.0 | 0.0 |
| **D-A** | 740 | 182 | 0.25 | 1362 | 76 | 0.06 | 3.9 | 3.9 | 0.3 |
| **D-A-D** | 671 | 176 | 0.26 | 1303 | 101 | 0.07 | 0.1 | 0.1 | 0.1 |
| **(D-A)$_n$** | 665 | 176 | 0.26 | 1254 | 101 | 0.06 | 0.1 | 0.0 | 0.1 |
| hexylated, geometry: BP86/Def2-SVP | | | | | | | | | |
| **A** | 808 | 179 | 0.22 | 1539 | 88 | 0.06 | 0.0 | 0.0 | 0.0 |
| **D-A** | 736 | 173 | 0.24 | 1457 | 91 | 0.06 | 2.3 | 2.3 | 0.2 |
| **D-A-D** | 688 | 170 | 0.25 | 1387 | 96 | 0.07 | 0.1 | 0.2 | 0.2 |
| **(D-A)$_n$** | 675 | 173 | 0.26 | 1384 | 87 | 0.06 | 0.2 | 0.0 | 0.2 |
| hexylated, geometry: B3LYP/6-31G** | | | | | | | | | |
| **A** | 818 | 177 | 0.22 | 1539 | 88 | 0.06 | 0.0 | 0.0 | 0.0 |
| **D-A** | 766 | 176 | 0.23 | 1457 | 91 | 0.06 | 1.7 | 1.7 | 0.0 |
| **D-A-D** | 726 | 174 | 0.24 | 1387 | 96 | 0.07 | 0.2 | 0.4 | 0.4 |
| **(D-A)$_n$** | 736 | 176 | 0.24 | 1384 | 87 | 0.06 | 0.1 | 0.2 | 0.3 |

1. Stoll, S.; Schweiger, A. EasySpin, a comprehensive software package for spectral simulation and analysis in EPR. *J. Magn. Reson.* **2006**, *178*, 42–55.
2. Matt, C.; Meyer, D.L.; Lombeck, F.; Sommer, M.; Biskup, T. TBT entirely dominates the electronic structure of the conjugated copolymer PCDTBT: Insights from time-resolved electron paramagnetic resonance spectroscopy. *Macromolecules* **2018**, *51*, 4341–4349.
3. Becke, A.D. Density–functional exchange–energy approximation with correct asymptotic behavior. *Phys. Rev. A* **1988**, *38*, 3098–3100.
4. Perdew, J.P. Density–functional approximation for the correlation energy of the inhomogeneous electron gas. *Phys. Rev. B* **1986**, *33*, 8822–8824.
5. Weigend, F.; Ahlrichs, R. Balanced basis sets of split valence, triple zeta valence and quadruple zeta valence quality for H to Rn: Design and assessment of accuracy. *Phys. Chem. Chem. Phys.* **2005**, *7*, 3297–3305.
6. Becke, A.D. Density-functional thermochemistry. III. The role of exact exchange. *J. Chem. Phys.* **1993**, *98*, 5648–5652.
7. Lee, C.; Yang, W.; Parr, R.G. Development of the Colle-Salvetti correlation-energy formula into a functional of the electron density. *Phys. Rev. B* **1988**, *37*, 785–789.
8. Petersson, G.A.; Bennett, A.; Tensfeldt, T.G.; Al-Laham, M.A.; Shirley, W.A.; Mantzaris, J. A complete basis set model chemistry. I. The total energies of closed-shell atoms and hydrides of the first-row elements. *J. Chem. Phys.* **1988**, *89*, 2193–2218.
9. Petersson, G.A.; Al-Laham, M.A. A complete basis set model chemistry. II. Open-shell systems and the total energies of the first-row atoms. *J. Chem. Phys.* **1991**, *94*, 6081–6090.
10. Neese, F. The ORCA program package. *Wiley Interdiscip. Rev.-Comput. Mol. Sci.* **2012**, *2*, 73–78.
11. Sinnecker, S.; Rajendran, A.; Klamt, A.; Diedenhofen, M.; Neese, F. Calculation of solvent shifts on electronic g-tensors with the conductor-like screening model (COSMO) and its self-consistent generalization to real solvents (direct COSMO-RS). *J. Phys. Chem. A* **2006**, *110*, 2235–2245.
12. Hanwell, M.D.; Curtis, D.E.; Lonie, D.C.; Vandermeersch, T.; Zurek, E.; Hutchison, G.R. Avogadro: an advanced semantic chemical editor, visualization, and analysis platform. *J. Cheminformatics* **2012**, *4*, 17.
13. Pettersen, E.F.; Goddard, T.D.; Huang, C.C.; Couch, G.S.; Greenblatt, D.M.; Meng, E.C.; Ferrin, T.E. UCSF Chimera—A visualization system for exploratory research and analysis. *J. Comput. Chem.* **2004**, *25*, 1605–1612.
14. Barone, V. Structure, Magnetic Properties and Reactivities of Open-Shell Species From Density Functional and Self-Consistent Hybrid Methods. In *Recent Advances in Density Functional Methods (Part I)*; Chong, D.P., Ed.; World Scientific: Singapore, 1995; Vol. 1, *Recent Advances in Computational Chemistry*, chapter 8, p. 287–334.
15. Hall, P.L.; Angel, B.R.; Jones, J.P.E. Dependence of spin Hamiltonian parameters $E$ and $D$ on labeling of magnetic axes: application to ESR of high-spin $Fe^{3+}$. *J. Magn. Reson.* **1974**, *15*, 64–68.
